# Correction: Circular RNA Is Expressed across the Eukaryotic Tree of Life

**DOI:** 10.1371/journal.pone.0095116

**Published:** 2014-04-08

**Authors:** The PLOS ONE Staff

## Notice of Republication

This article was republished on March 20, 2014, to correct the ordering of figures. The publisher apologizes for the error. Please download this article again to view the correct version. The originally published, uncorrected article and the republished, corrected article are provided here for reference.

## Supporting Information

File S1 Originally published, uncorrected article.
(PDF)

File S2 Republished, corrected article.
(PDF)
