# Supplementary figures and images for: Circular RNA Is Expressed across the Eukaryotic Tree of Life
Source: PLoS One. 2014 Mar 7;9(3):e90859. doi: 10.1371/journal.pone.0090859 (PMC3946582; doi:10.1371/journal.pone.0090859)

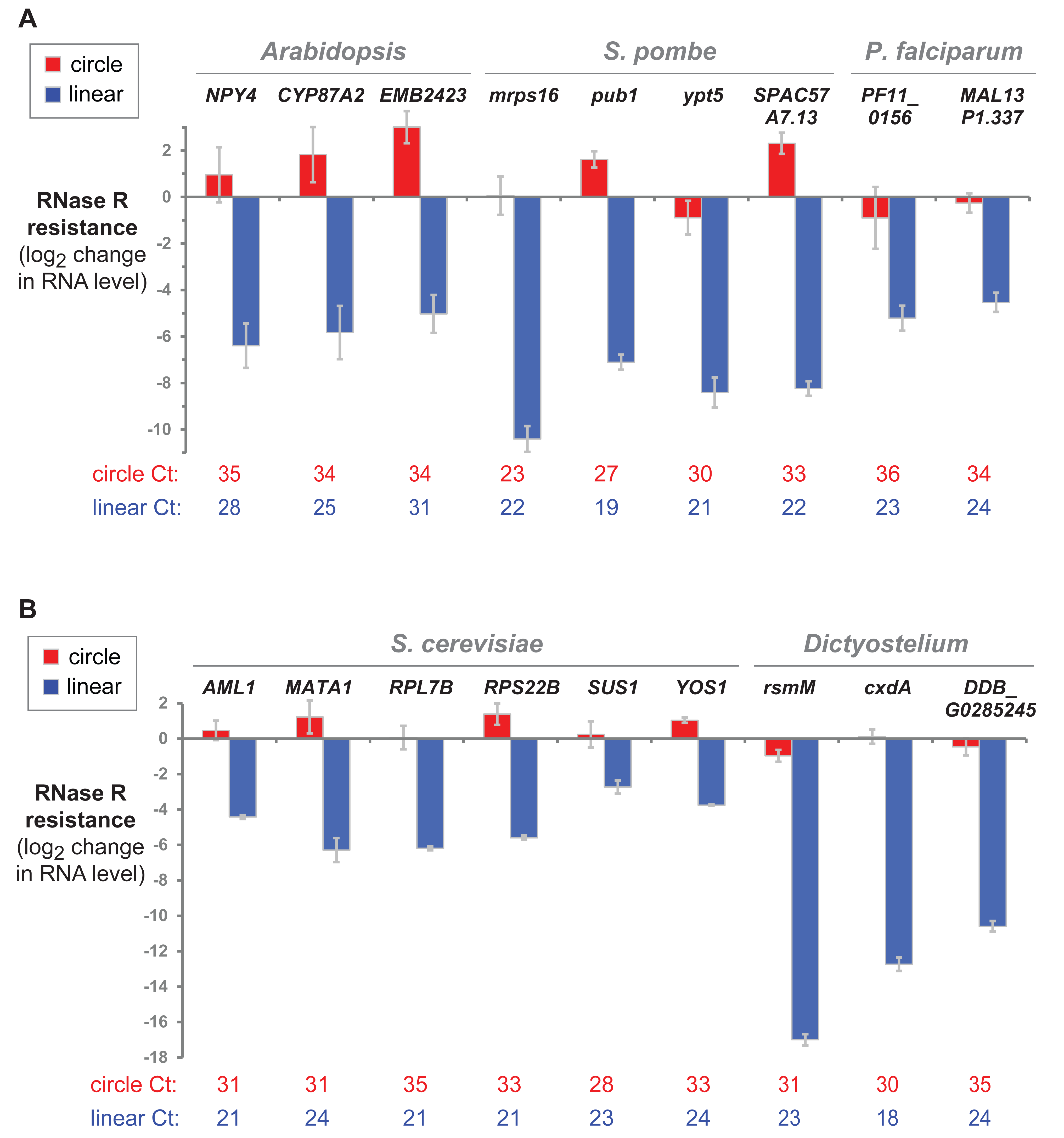

Supplement: Figure S1 — RNase R resistance of circular and linear isoforms. a) Quantitation of RNase R resistance. Plotted here is the RNase R resistance of each isoform (the log2 fold-change in RNA abundance with RNase R treatment), measured by quantitative RT-PCR and taken as ΔCt = Ct(mock-treatment) – Ct(RNase R-treatment). All linear isoforms were sensitive to RNase R, showing a greater than 32-fold drop in abundance after RNase R treatment (ΔCt <−5). Circular isoforms show no significant decrease in abundance with RNase R treatment, and in many cases the signal increases (see main text). The absolute Ct for mock-treated RNA is also given, as an indicator of the comparative abundance of circular and linear isoforms. For S. pombe, data shown here is for exponential growth. b) Quantitation of RNase R resistance for two additional species, Dictyostelium discoideum and S. cerevisiae. Format is the same as a). (TIF) [file pone.0090859.s001.tif]
