# Supplementary material for: Circular RNA Is Expressed across the Eukaryotic Tree of Life
Source: PLoS One. 2014 Mar 7;9(3):e90859. doi: 10.1371/journal.pone.0090859 (PMC3946582; doi:10.1371/journal.pone.0090859)
Supplement: Text S1 — PCR primers, Sanger sequencing. 1) List of primers used in PCR and qPCR. 2) Sanger sequences of detected circular RNA isoforms. (DOC) [file pone.0090859.s002.doc]

**PRIMER LIST**

| **gene** | **primer** | **sequence** | **product size (junction)** |
| --- | --- | --- | --- |
| ***Schizosaccharomyces pombe*** | | | |
| **mrps16** | circle-forward | GAGCACAACCATCCGACAC | 128 bp (1-1) |
|  | circle-reverse | CGAGGAATGGAGTCCTGAGA |  |
|  | linear-forward | ACACCGTCCGCTCTTTAGC | 231 bp (1-2) |
|  | linear-reverse | GGAAGGGTAAAATCGGTCAA |  |
| **ypt5** | circle-forward | AAGGAACTTCAGCGTCAAGC | 123 bp (7-6) |
|  | circle-reverse | CGACAATAGCGCAATTAGCAT |  |
|  | linear-forward | AAGGAACTTCAGCGTCAAGC | 206 bp (7-8) |
|  | linear-reverse | AAGGGAAGCTTTTTGGCAAT |  |
| **pub1** | circle-forward | ACAGTGGATGGTGAGCAAAC | 138 bp (2-2) |
|  | circle-reverse | GCAAACGGGTCAGGAAAACG |  |
|  | linear-forward | ACCCGTTTGCGGTACTAACA | 207 bp (2-3) |
|  | linear-reverse | GCACATCTCCAACACGAAGA |  |
| **SPAC57A7.13** | circle-forward | CGACGGTATTGATGTGTCTGTAC | 209 bp (2-2) |
|  | circle-reverse | CTTCCATCCAATCCTTAGAATCCTG |  |
|  | linear-forward | CGACGGTATTGATGTGTCTGTAC | 205 bp (1-2) |
|  | linear-reverse | GGGTACATCACTTACATCCATGTC |  |
| ***Plasmodium falciparum*** | | | |
| **MAL13P1.337** | circle-forward-D | CAGCAGACGAAATTCCCAAG | 308 bp (4-2) |
|  | circle-reverse-D | AAATTGGTGTTTTAATATTTGGAAGTG |  |
|  | circle-forward-E | GAAATTCGAGCGGAGTTTGA | 177 bp (4-2) |
|  | circle-reverse-E | AAGGCTTGGGAATTTCGTCT |  |
|  | linear-forward | AAAATACGGCATCTATGTCAACAG | 165 bp (1-2) |
|  | linear-reverse | AAGGCTTGGGAATTTCGTCT |  |
| **PF11_0156** | circle-forward | TGCAAGAAGAATACGATGAGGA | 209 bp (4-4) |
|  | circle-reverse | TGCATTTCCCTTACTTTTATTCA |  |
|  | linear-forward | TGCAAGAAGAATACGATGAGGA | 188 bp (4-5) |
|  | linear-reverse | ATCAATAACCTCGCCAACCA |  |
| ***Arabidopsis thaliana*** | | | |
| **NPY** | circle-forward | AGAACTTCCTCTCTCTCGCTGA | 124 bp (3-3) |
|  | circle-reverse | TTTCTGCAAGAATCCGCTTT |  |
|  | linear-forward | TGACCAAGCTACATCGAGCA | 699 bp (3-4) |
|  | linear-reverse | TTGGCCCAATTACAAAGGTC |  |
| **CYP87A2** | circle-forward | CATGGGAAGAATACAAGTCAATG | 323 bp (5-4) |
|  | circle-reverse | TTCCCGGTTTCTCAGAATTG |  |
|  | linear-forward | TCTGAGAAACCGGGAAGATG | 132 bp (5-6) |
|  | linear-reverse | AACGCCTTTCTGAAGATTGC |  |
| **EMB2423** | circle-forward | AAGCAGTGACAGATCCGTATG | 616 bp (8-6) |
|  | circle-reverse | GTCCAACTGAAATGTATCCACC |  |
|  | linear-forward | ATATTGGACGACAGGGCTTC | 554 bp (6-8) |
|  | linear-reverse | GCTAGTCGCTGCGTTGTTTC |  |

| ***Saccharomyces cerevisiae*** | | | |
| --- | --- | --- | --- |
| **gene** | **primer** | **sequence** | **product size (junction)** |
| **AML1**  (YGR001C) | circle-forward | AACCACTGGACTTCAGTGATGA | 179 (2-2) |
|  | circle-reverse | CGTCGTAAAGCTTTTGAAAGGCTTC |  |
|  | linear-forward | CGACTCCGACTCCGATTATG | 113 (1-2) |
|  | linear-reverse | CGTCGTAAAGCTTTTGAAAGGCTTC |  |
| **MATa1** = HMRA1  (YCR097W) | circle-forward | GAAAGCAAAGCCTTAATTCCAAGG | 196 (2-2) |
|  | circle-reverse | TTTCCCTTTGGGCTCTTCTCT |  |
|  | linear-forward | TGGCGGAAAACATAAACAGA | 202 (1-2) |
|  | linear-reverse | TTTCCCTTTGGGCTCTTCTCT |  |
| **RPL7A**  (YGL076C) | circle-forward | CTGCTGAAAGAGCTGCTCGTA | 97 (2-2; not observed) |
|  | circle-reverse | CAGCGACTTGTTCAGCAGTCT |  |
|  | linear-forward | CTGCTGAAAGAGCTGCTCGTA | 127 (2-3) |
|  | linear-reverse | GCATCACGCTTAGCTTGAATGATG |  |
| **RPL7B**  (YPL198W) | circle-forward | CAGAGAGAGCTGCCCGTAAA | 97 (2-2) |
|  | circle-reverse | CTGCAGCAATTTGTTCTGCAGTC |  |
|  | linear-forward | CAGAGAGAGCTGCCCGTAAA | 128 (2-3) |
|  | linear-reverse | CTTGGCATCACGCTTAGCTTG |  |
| **RPS22B**  (YLR367W) | circle-forward | CGTCAGGTTCTATTGAGACCTTC | 135 (2-2) |
|  | circle-reverse | CGGTCTTTTCGGCGTTATTAATGG |  |
|  | linear-forward | CGTCAGGTTCTATTGAGACCTTC | 134 (2-3) |
|  | linear-reverse | CCGTTCAGTTGGACGACAATC |  |
| **SUS1**  (YBR111W-A) | circle-forward | GGGTGGATAAAGTCAAAGATTTGAC | 139 (2-2) |
|  | circle-reverse | ACCTTCTTGAAGTAGTCTGGCT |  |
|  | linear-forward | GGATACTGCGCAATTAAAGAGTC | 103 (1-2) |
|  | linear-reverse | ACCTTCTTGAAGTAGTCTGGCT |  |
| **TAD3**  (YLR316C) | circle-forward | GATCCAAGAGACTCGAAGAAAGT | 62 (2-2; not observed) |
|  | circle-reverse | CTCTTGGATCTATTCTTATACTCCATAC |  |
|  | linear-forward | CGGGATAATAGAAAATCGGCTTCTG | 98 (1-2) |
|  | linear-reverse | CTCTTGGATCTATTCTTATACTCCATAC |  |
| **VMA9**  (**YCL005W-A**) | circle-forward | GGATAATGGCTCCAAAGAACAAC | 82 (2-2; not observed) |
|  | circle-reverse | CAGAACAGCACGGACATGG |  |
|  | linear-forward | GGATAATGGCTCCAAAGAACAAC | 108 (2-3) |
|  | linear-reverse | GATGGAGCTGGCAAAGGAAC |  |
| **YOS1**  (YER074W-A) | circle-forward | CCACGAAGTCCAAGGTTGTTC | 99 (2-2) |
|  | circle-reverse | CCTGACCAAAAACAGGTGTCTC |  |
|  | linear-forward | CCACGAAGTCCAAGGTTGTTC | 122 (2-3) |
|  | linear-reverse | CGTACGCTTAGTTCATCCCAATAG |  |

| **gene** | **primer** | | **sequence** | **product size (junction)** |
| --- | --- | --- | --- | --- |
| ***Dictyostelium discoideum*** | | | | |
| **rsmM** | circle-forward | GCATTAGCAAGTACAAAGAGGGAT | | 197 (5-4) |
|  | circle-reverse | CTTTGAATTTCGGTGTGCCATTGAAC | |  |
|  | linear-forward | GCATTAGCAAGTACAAAGAGGGAT | | 130 (5-7) |
|  | linear-reverse | GCTTCCCCTTGTTTGACGTG | |  |
| **cxdA** | circle-forward | CCATCAGAACTCTCAAAACAATCTGGTT | | 208 (2-2) |
|  | circle-reverse | CGAATTCTGGTGAACCTACTTTGATG | |  |
|  | linear-forward | CGTTCAGCTACTAAAGCTTTCCAAAC | | 154 (1-2) |
|  | linear-reverse | CGAATTCTGGTGAACCTACTTTGATG | |  |
| **DDB_G0285245** | circle-forward | CTTTCATCACCAAGACAATCTGTATCTAC | | 263 (2-2) |
|  | circle-reverse | GTTTGTGGTGACAATGGAGAAGAG | |  |
|  | linear-forward | GCAAAATTTATTTAGAATCAATCTCTTATGACC | | 193 (1-2) |
|  | linear-reverse | GTTTGTGGTGACAATGGAGAAGAG | |  |

For *Arabidopsis* and *P. falciparum*, different primer-sets were used for qPCR, shown below, than for the conventional PCRs. For the other species, the same primers were used for both.

| **gene** | **primer** | **sequence** | **product size (junction)** |
| --- | --- | --- | --- |
| ***Arabidopsis thaliana*** | | | |
| **NPY** | circle-forward | AGAACTTCCTCTCTCTCGCTGA | 124 bp (3-3) |
|  | circle-reverse | TTTCTGCAAGAATCCGCTTT |  |
|  | linear-forward | GACACTCTCTAGCTTCCCAAGAC | 163 bp (3-4) |
|  | linear-reverse | CTGTACCGCATGTTCACACG |  |
| **CYP87A2** | circle-forward | CATGGGAAGAATACAAGTCAATG | 323 bp (exon 5) |
|  | circle-reverse | TTCCCGGTTTCTCAGAATTG |  |
|  | linear-forward | TGCTTCAAGAGAGGCGTGAG | 278 bp (4-5) |
|  | linear-reverse | TTCCCATGTAAGTCCAGAGTCTGC |  |
| **EMB2423** | circle-forward | GAAACAACGCAGCGACTAGC | 160 bp (8-6) |
|  | circle-reverse | AATTGGCCTCAGTGCAGGAAG |  |
|  | linear-forward | TCTAGCAATGGAAGTCTCAAGC | 178 bp (5-6) |
|  | linear-reverse | AATTGGCCTCAGTGCAGGAAG |  |
| ***Plasmodium falciparum*** | | | |
| **MAL13P1.337** | circle-forward | GAAGAAATTCGAGCGGAGTTTG | 94 bp (4-2) |
|  | circle-reverse | GGAAGTGGTATAGTGTCTTCTTCTG |  |
|  | linear-forward | AAAATACGGCATCTATGTCAACAG | 165 bp (1-2) |
|  | linear-reverse | AAGGCTTGGGAATTTCGTCT |  |
| **PF11_0156** | circle-forward | GATAATTGGAATGACTCAGAGGGA | 130 bp (4-4) |
|  | circle-reverse | CTTCTTCATTTTCAAGAGGACTCTTC |  |
|  | linear-forward | TGCAAGAAGAATACGATGAGGA | 188 bp (4-5) |
|  | linear-reverse | ATCAATAACCTCGCCAACCA |  |

**SANGER SEQUENCING**

***Schizosaccharomyces pombe***

pombe PCR products were all sequenced directly. In all cases, the sequencing showed rolling circle repeats; for simplicity, only the minimal product sequence is shown. Exon junctions are indicated by change of case.

***mrps16***

GAGCACAACCATCCGACACCGTCCGCTCTTTAGCTGAAAAGgaaagcacctcaagccaaacctatcgaaaccatcggcacttttgatcccatccctaagaagattgattctcaggactccattcctcg

***ypt5***

AAGGAACTTCAGCGTCAAGCTCCAGAGGGTATTGTTATAGCTTTGGCCGGCAACAAGCTGGATCTTGCCCAAGAACGTAGAGCTGTTGAGAAAGCTatcgaaatgctaattgcgctattgtcg

***pub1***

ACAGTGGATGGTGAGCAAACACATACCACAACTGCTATCAAAAAGACGTTAAACCCTTATTGGAATGAGACTTTTGAAGTttgttgctgctgatggcctttacaaacgagatgtgtttcgttttcctgacccgtttgc

***SPAC57A7.13***

CGACGGTATTGATGTGTCTGTACGTTTTTCACGTGCGGCCAGAGAGCAGATTGAGGGTTGGTGTTGCCAAAATattgaacttgcgttgtacgccacgggtgctaaagtttctactgttacactaataagagagaaagaaacacaaaaatctcgctgttttgccttcgctaaatttgtttccttacaggattctaaggattggatggaag

***Plasmodium falciparum***

***PF11_0156***

**bcR60** (directly sequenced PCR product) is a exon 4 circle. Exon junction indicated by change of case.

TGCAAGAAGAATACGATGAGGATAATGATGATTTTGATATGTTTTCCTGTGTACAAGCAAATAAAAAAAGAAAAGTTGAAAAAGTACATATAACTGATTATTACACAACAGGAAATAATGCAAATTTGTCAGATAATTGGAATGACTCAGAGGGATATTACAAGaatgtggatgatagtttatatgtgaataaaagtaagggaaatgca

**bc#2** (TOPO-clone) is a 3-4 circle. Below, exon3 in black and lowercase, exon4 sequence is in green.

TGCAAGAAGAATACGATGAGGATAATGATGATTTTGATATGTTTTCCTGTGTACAAGCAAATAAAAAAAGAAAAGTTGAAAAAGTACATATAACTGATTATTACACAACAGGAAATAATGCAAATTTGTCAGATAATTGGAATGACTCAGAGGGATATTACAAGtgatgcggcctgcatttttgcacccaacaatgatgttattgaagatacgcgctcatcactttcatcagatcatgaaattatagaagaaaaacaaaataaagaaaaaccagaagcagtaaaagagtgtagtgatttgtataatgatttaaaaaaaaaaattgatgaagaaaaggccaaaattaggtcatttataatcaaacagaaagaattacatgaaagattaaaaatgaatgtggatgatagtttatatgtgaataaaagtaagggaaatgca

**PF11_0156 "bc4"** is direct sequencing of qPCR product. It represents an exon 4 circle.

GATAATTGGAATGACTCAGAGGGATATTACAAGaatgtggatgatagtttatatgtgaataaaagtaagggaaatgcagatacacataataatttaactaataagaagagtcctcttgaaaatgaagaag

***MAL13P1.337***

TOPO-clones from PCR primer-pairs D and E.

Below, exon 1 sequence is in blue, exon 2 in red, exon 3 in black, exon 4 sequence is in green.

Note that in the current genome annotation, "exon 1" begins at the ATG start codon;the true gene likely contains additional 5' exonic sequence.

**D#8** is an exon 1a-2 circle, where exon 1 has alternative (shorter) 5' end, missing sequence is given in brackets and italic lowercase. There are rolling repeats. (400 bp)

CAGCAGACGAAATTCCCAAGCCTTTGATTACATCGAACT‑ACAAGAC[*atgaaaaatgataagataaaattag*]TAAGTTTTGAGGGTGATGAATTTATAGTTGAcAAAAATACGGCATCTATGTCAACAGTAATAATGAATATATTGGAAGTTATGACAGCAGAAGAAGACACTATACCACTTCCAAATATTAAAACACCAATTTTAAAAAAAATAATTGAATATATGGAATATCATATTAATAACCCAGCAGACGAAATTCCCAAGCCTTTGATTACATCGAACTTACAAGAC[*atgaaaaatgataagataaaattag*]TAAGTTTTGAGGGTGATGAATTTATAGTTGATAAAAATACGGCATCTATGTCAACAGTAATAgTGAATATATTGGAAGTTATGACAGCAGAAGAAGACACTATACCACTTCCAAATATTAAAACACCAATTT

**E#2** is an exon 2-3-4 circle. (177 bp)

GAAATTCGAGCGGAGTTTGATATTGTTAACGATTTTACAAGAGAAGAAGAAAAACAATTATGACAGCAGAAGAAGACACTATACCACTTCCAAATATTAAAACACCAATTTTAAAAAAAATAATTGAATATATGGAATATCATATTAATAACCCAGCAGACGAAATTCCCAAGCCTT

**E#4, E#5** are an exon 1b-2-3-4 circle, where exon 1 has alternative (shorter) 5' end, missing sequence is given in brackets and italic lowercase. There are rolling repeats. (265 bp)

GAAATTCGAGCGGAGTTTGATATTGTTAACGATTTTACAAGAGAAGAAGAAAAACAA[*atgaaaaatgataag*]ATAAAATTAGTAAGTTTTGAGGGTGATGAATTTATAGTTGATAAAAATACGGCATCTATGTCAACAGTAATAATGAATATATTGGAAGTTATGACAGCAGAAGAAGACACTATACCACTTCCAAATATTAAAACACCAATTTTAAAAAAAATAATTGAATATATGGAATATCATATTAATAACCCAGCAGACGAAATTCCCAAGCCTT

**Da#1** is a 2-2 circle; with rolling repeats. exon-exon junctions are indicated by change of case. (530 bp)

cagcagacgaaattcccaagcctttgattacatcgaacttacaagacTTATGACAGCAGAAGAAGACACTATACCACTTCCAAATATTAAAACACCAATTTTAAAAAAAATAATTGAATATATGGAATATCATATTAATAACCCAGCAGACGAAATTCCCAAGCCTTTGATTACATCGAACTTACAAGACttatgacagcagaagaagacactataccacttccaaatattaaaacaccaattttaaaaaaaataattgaatatatggaatatcatattaataacccagcagacgaaattcccaagcctttgattacatcgaacttacaagacTTATGACAGCAGAAGAAGACACTATACCACTTCCAAATATTAAAACACCAATTTTAAAAAAAATAATTGAATATATGGAATATCATATTAATAACCCAGCAGACGAAATTCCCAAGCCTTTGATTACATCGAACTTACAAGACttatgacagcagaagaagacactataccacttccaaatattaaaacaccaattt

**Da#1** is an exon 1a-2 circle. (179 bp)

CAGCAGACGAAATTCCCAAGCCTTTGATTACATCGAACTTACAAGACTAAGTTTTGAGGGTGATGAATTTATAGTTGATAAAAATACGGCATCTATGTCAACAGTAATAATGAATATATTGGAAGTTATGACAGCAGAAGAAGACACTATACCACTTCCAAATATTAAAACACCAATTT

**Ea#10** is an exon 2-3-4 circle; with rolling repeats. (527 bp)

GAAATTCGAGCGGAGTTTGATATTGTTAACGATTTTACAAGAGAAGAAGAAAAACAATTATGACAGCAGAAGAAGACACTATACCACTTCCAAATATTAAAACACCAATTTTAAAAAAAATAATTGAGTATATGGAATATCATATTAATAACCCAGCAGACGAAATTCCCAAGCCTTTGATTACAACGAACTTACAAGACGTTGTATCTTCATGGGATTTTGATTTTGTAAATACTGATAAGGAGACCCTTTACGAACTAATCGAAGCGTCAAACTATCTTGATATTAAACCTCTTCTTGATTTGACTTGCGGAAAAATCGCTTCAATGATGAAAGATAAAACTACCGAAGAAATTCGAGCGGAGTTTGATATTGTTAACGATTTTACGAGAGAAGAAGAAAAACAATTATGACAGCAGAAGAAGACACTATACCACTTCCAAATATTAAAACACCAATTTTAAAAAAAATAATTGAATATATGGAATATCATATTAATAACCCAGCAGACGAAATTCCCAAGCCTT

**Eb#9** is an exon 1b-2-3-4 circle, where exon 1 has alternative (shorter) 5' end, missing sequence is given in brackets and italic lowercase. There are rolling repeats. (703 bp)

GAAATTCGAGCGGAGTTTGATATCGTTAACGATTTTACAAGAGAAGAAGAAAAACAA[*atgaaaaatgataag*]ATAAAATTAGTAAGTTTTGAGGGTGATGAATTTATAGTTGATAAAAATACGGCATCTATGTCAACAGTAATAATGAATATATTGGAAGTTATGACAGCAGAAGAAGACACTATACCACTTCCAAATATTAAAACACCAATTTTAAAAAAAATAATTGAATATATGGAATATCATATTAATAACCCAGCAGACGAAATTCCCAAGCCTTTGATTACATCGAACTTACAAGACGTTGTATCTTCATGGGATTTTGATTTTGTAAATACTGATAAGGAGACCCTTTACGAACTAATCGAAGCGTCAAACTATCTTGATATTAAACCTCTTCTTGATTTGACTTGCGGAAAAATCGCTTCAATGATGAAAGATAAAACTACCGAAGAAATTCGAGCGGAGTTTGATATTGTTAACGATTTTACAAGAGAAGAAGAAAAACAAATAAAATTAGTAAGTTTTGAGGGTGATGAATTTATAGTTGATAAAAATACGGCATCTATGTCAACAGTAATAATGAATATATTGGAAGTTATGACAGCAGAAGAAGACACTATACCACTTCCAAATATTAAAACACCAATTTTAAAAAAAATAATTGAATATATGGAATATCATATTAATAACCCAGCAGACGAAATTCCCAAGCCTT

**MAL13P1.337 "E3"** Direct sequencing of qPCR product. This represents an exon 1b-2-3 circle; color coding as above.

GAAGAAATTCGAGCGGAGTTTGATATCGTTAACGATTTTACAAGAGAAGAAGAAAAACAA[*atgaaaaatgataag*]ATAAAATTAGTAAGTTTTGAGGGTGATGAATTTATAGTTGATAAAAATACGGCATCTATGTCAACAGTAATAATGAATATATTGGAAGTTATGACAGCAGAAGAAGACACTATACCACTTCC

***Arabidopsis thaliana***

***NPY4***

**F1** (directly sequenced PCR product) is a 3-3 circle. Exon-exon junction indicated by change of case.

AGAACTTCCTCTCTCTCGCTGAGACACTCTCTAGCTTCCCAAGACACTCTCACGACGTGTTATATCGCGCAATTGACATGTTTCTAAAGttcccattgctgtcgaaaagcggattcttgcagaaa

***EMB2423***

**G1** (directly sequenced PCR product) is an exon 6-7a-8a circle, where exon 7 has an alternative (shorter) 3' end, and exon 8 has an alternative (longer) 5' end. The sequence missing in exon 7 is given in brackets and italic lowercase; the additional sequence in exon 8 is given in lowercase.

Below, exon 6 sequence is in blue, exon 7 in red, exon 8 in black.

AAGCAGTGACAGATCCGTATGCTGTTGAGAAAATGGCTGAACAACTTTTACATCAGCTATATGCTGAGCATCCAAGTGATGTTGAAGCTTTCTGGACTATTTGGACTTTGTTTCATCGCAATGTAATACATCAGGCATCAGTGAGGCAA[*gcaaaatgttttctatggcagcttgattctttctttagatacccctttttcttctttcatttccatcctaatgctgtgaaacaatgcg*]gtcaatattcgttgacaaatttttgctatggaaagtgtttcctatacgttgtcttcgatggatcttacaattttca‑TTCTTGAGTGCCCACCGGTTACTAATACACTTGCTAAAGGTGATGTCACACAAGGACTTCTTGAAACAACGCAGCGACTAGCCTCAGTTTGGTCAAAACGGGAGTTTCTGCAGTCCGTCCAATTGGAGCAGCAAGCTTCTTGTACTTCAAGCACATTACCAATCAGCTTCTACAAATATTGGACGACAGGGCTTCCTGCACTGAGGCCAATTGCACAGTTATCGTTTTATCATTTGTCGGGGAAGTTTTTTCCCGCATCTGTCGACGTGGATTATCAGATTTGCTGTTAAGTGAAGTAACACCTCATGTCCTAGCTCAAGTTAGGAGGTTACTAAACTCAAAGATAGGTGCTATTGAGGTGGATACATTTCAGTTGGAC

***CYP87A2***

TOPO-clones from PCR product.

Below, exon 4 sequence is in blue, exon 5 in red and the longer extension of exon 5 in red lower case, exon 6 in purple.

**H1** is an exon 5a circle, where exon 5 has an alternative (longer) 3' end; with rolling repeats. (540 bp)

ATGGGAAGAATACAAGTCAATGACTTACACATTTCAGgttgctttaaagttttatcaaaGAAGAACATGAGACAATTCTGAGAAACCGGGAAGATGCAGACTCTGGACTTACATGGGAAGAATACAAGTCAATGACTTACACATTTCAGgttgctttaaagttttatcaaaAGAAGAACATGAGACAATTCTGAGAAACCGGGAAGATGCAGACTCTGGACTTACATGGGAAGAATACAAGTCAATGACTTACACATTTCAGgttgctttaaagttttatcaaaGAAGAACATGAGACAATTCTGAGAAACCGGGAAGATGCAGACTCTGGACTTACATGGGAAGAATACAAGTCAATGACTTACACATTTCAGgttgctttaaagttttatcaaaGAAGAACATGAGACAATTCTGAGAAACCGGGAAGATGCAGACTCTGGACTTACATGGGAAGAATACAAGTCAATGACTTACACATTTCAGgttgctttaaagttttatcaaaGAAGAACATGAGACAATTCTGAGAAACCGGGAA

**H2** is an exon 5a circle, where exon 5 has an alternative (longer) 3' end; with rolling repeats. (204 bp)

ATGGGAAGAATACAAGTCAATGACTTACACATTTCAGgttgctttaaagttttatcaaaGAAGAACATGAGACAATTCTGAGAAACCGGGAAGATGCAGACTCTGGACTTACATGGGAAGAATACAAGTCAATGACTTACACATTTCAGgttgctttaaagttttatcaaaGAAGAACATGAGACAATTCTGAGAAACCGGGAA

**H3** is an exon 4-5a circle, where exon 5 has an alternative (longer) 3' end. (347 bp)

ATGGGAAGAATACAAGTCAATGACTTACACATTTCAGgttgctttaaagttttatcaaaGGTAGGGCAAAGGCAATGAAAATGTTGAGGAATATGCTTCAAGAGAGGCGTGAGAACCGTCGGAAGAATCCAAGTGATTTCTTTGATTATGTTATTGAAGAGATTCAGAAAGAAGGGACAATTCTGACAGAAGAGATTGCACTGGATTTGATGTTTGTCTTGCTATTTGCCAGTTTTGAAACAACTTCTCTGGCTCTAACTTTAGCCATCAAGTTTCTTTCAGATGACCCTGAAGTCCTAAAGCGTTTAACGGAAGAAGAACATGAGACAATTCTGAGAAACCGGGAA

**H4** is an exon 4-5-6a circle, where exon 6 has an alternative (shorter) 3' end, missing sequence is given in brackets and italic lowercase. (381 bp)

ATGGGAAGAATACAAGTCAATGACTTACACATTTCAGTTCATAAACGAAACCGCGAGACTAGCAAATATAGTTCCTGCAATCTTCAGAAAGGC[*gttgagagatataaaattcaaag*]GGTAGGGCAAAGGCAATGAAAATGTTGAGGAATATGCTTCAAGAGAGGCGTGAGAACCGTCGGAAGAATCCAAGTGATTTCTTTGATTATGTTATTGAAGAGATTCAGAAAGAAGGGACAATTCTGACAGAAGAGATTGCACTGGATTTGATGTTTGTCTTGCTATTTGCCAGTTTTGAAACAACTTCTCTGGCTCTAACTTTAGCCATCAAGTTTCTTTCAGATGACCCTGAAGTCCTAAAGCGTTTAACGGAAGAAGAACATGAGACAATTCTGAGAAACCGGGAA

**H5** is an exon 4-5 circle. (325 bp)

ATGGGAAGAATACAAGTCAATGACTTACACATTTCAGGGTAGGGCAAAGGCAATGAAAATGTTGAGGAATATGCTTCAAGAGAGGCGTGAGAACCGTCGGAAGAATCCAAGTGATTTCTTTGATTATGTTATTGAAGAGATTCAGAAAGAAGGGACAATTCTGACAGAAGAGATTGCACTGGATTTGATGTTTGTCTTGCTATTTGCCAGTTTTGAAACAACTTCTCTGGCTCTAACTTTAGCCATCAAGTTTCTTTCAGATGACCCTGAAGTCCTAAAGCGTTTAACGGAAGAAGAACATGAGACAATTCTGAGAAACCGGGAA

***Dictyostelium discoideum***

***rsmM***

Directly sequenced PCR product, is a 5-4 circle. Exon-exon junction indicated by change of case.

gcattagcaagtacaaagagggatttacctcctgtagttgacccatcattagctcaaAAATCAAGAGCAATTCAATTTGGGAAAAAGCAGATATTAGTAAAATTTtttgacacagcaggccaagaaagatttagaaccatttcaaaatcattttattcaaacacagatataattattcttacatatgaccaaaacaatcaatcaacatttgatcatcttgttcaatggcacaccgaaattcaaag

***cxdA***

Directly sequenced PCR product, is a 2-2 circle. Exon-exon junction indicated by change of case.

ccatcagaactctcaaaacaatctggtttaagtgatgccctcctcgatgacccaatcctccacatttgtgtcatcaaatacaacaaaaacattgtccaaagAGGATTTTTACAAACTACCCTTAAAAACGTCCTCTTCCCAACTGAAAGACAATTAAGACGTCAATACTTAGCTGATAATCACATCAAAGTAGGTTCACCAGAATTCG

***DDB_G0285245***

Directly sequenced PCR product, is a 2-2 circle. Exon-exon junction indicated by change of case.

ctttcatcaccaagacaatctgtatctacaaaatcaaccccattaaaatcaaactctaataataataataataattcatcaaaccatattagtttcttagacggtacatcacttAAATCAAAATTCAAGAGTAATACTCCATACAAAGCACATCGTAAAACTACATCAGCATTGGTTAGAAATGCAGATGCAGCATTGTATGATATAACAAATTCTCCACCACAATCAACTAGTAAATACTCTTCTCCATTGTCACCACAAAC

***Saccharomyces cerevisiae***

***AML1*** *(YGR001C)*

Directly sequenced PCR product, is an 2-2 circle. Exon-exon junction indicated by change of case.

AACCACTGGACTTCAGTGATGAAATTAAAGGAAAAGTTGATAGATTGTTAATTGACCCACCTTTTTTAAATGAAGATTGTCAAACAAAGTgacactttctgctaatgccctcgctgcccttgaagaattcaaaagagaggaacaacaacatcaagaagcctttcaaaagctttacgacg

***MATA1***

Directly sequenced PCR product, is a 2-2 circle. Exon-exon junction indicated by change of case.

GAAAGCAAAGCCTTAATTCCAAGGAAAAAGAAGAAGTTGCAAAGAAATGTGGCATTACTCCACTTCAAGTAAGAGTTTGGtttataatggaaagtaatttgactaaagtagagcaacatacattacacaaaaatatttctaacaataggttagaaatataccaccacattaaaaaagagaagagcccaaagggaaa

***RPL7B***

Directly sequenced PCR product, is a 2-2 circle. Exon-exon junction indicated by change of case.

TGCAGAACAAATTGCTGCAGAGAGAGCTGCCCGTAAAGCCaaaaatcttgactcctgaatctcaattgaagaagtctaaggctcaacaaaagactgctgaacaagtcgctg

***RPS22B***

TOPO-clones from qPCR. The major clone sequenced is a 2-2 circle. Exon-exon junction indicated by change of case.

CGTCAGGTTCTATTGAGACCTTCTTCCAAGGTTATCATCAAGTTTTTACAAGTTATGCAAAAGCATGgtaaaaaatgactcgctcttccgttttagctgatgctttgaatgccattaataacgccgaaaagaccg

There is also a minor population that splices to a cryptic acceptor site 7 nt upstream of the annotated acceptor site of the first intron. The additional bases are underlined.

CGTCAGGTTCTATTGAGACCTTCTTCCAAGGTTATCATCAAGTTTTTACAAGTTATGCAAAAGCATG

taattaggtaaaaaatgactcgctcttccgttttagctgatgctttgaatgccattaataacgccgaaaagaccg

***SUS1***

Directly sequenced PCR product, is an 2-2 circle. Exon-exon junction indicated by change of case.

GGGTGGATAAAGTCAAAGATTTGACCAAATCAGAGATGAACATAAATGAATCTACGAACTTTACCCAAATTTTATCCACCGTAGAACCCAAAGCATTAGaatttcaaacgaactaaaagccagactacttcaagaaggt

***TAD3***

TOPO-clones from qPCR. All clones contain sequence from apparent cryptic splicing to one of two acceptor sites in exon 1. Exon 1 sequences are shown in lowercase. Some clones contain more than one repeat, generated either due to rolling circle reverse transcription or to template-switching during PCR.

• qRT-PCR Ct values (mock-treated/RNase R-treated): circle = 34.7/32.6; linear = 24.3/26.6.

*TAD3 #1:*

GATCCAAGAGACTCGAAGAAAGTAATAGAaaatttcaaagatgtaaatacgcccaaactaaTCAATGTATGGAGTATAAGAATAGATCCAAGAG

*TAD3 #4:*

GATCCAAGAGACTCGAAGAAAGTAATAGAatgtaaatacgcccaaactaaTCAATGTATGGAGTATAAGAATCGATCCAAGAGACTCGAAGAAAGTAATAGAaaatttcaaagatgtaaatacgcccaaactaaTCAATGTATGGAGTATAAGAATAGATCCAAGAG

*TAD3 #3:*

GATCCAAGAGACTCGAAGAAAGTAATAGAaaatttcaaagatgtaaatacgcccaaactaaTCAATGTATGGAGTATAAGAATAGATCCAAGAGACTCGAAGAAAGTAATAGAaaatttcaaagatgtaaatacgcccaaactaaTCAATGTATGGAGTATAAGAATTGATCCAAGAGACTCGAAGAAAGTAATAGAatgtaaatacgcccaaactaaTCAATGTATGGAGTATAAGAATAGATCCAAGAG

*TAD3 #9:*

GATCCAAGAGACTCGAAGAAAGTAATAGAatgtaaatacgcccaaactaaTCAATGTATGGAGTATAAGAATAGATCCAAGAG

***YOS1***

TOPO-clones from qPCR. Some clones contain more than one repeat, generated either due to rolling circle reverse transcription or to template-switching during PCR; exon 2 sequence is indicated in blue or black, to help demarcate repeats. Dashes indicate missing bases (all within reverse primer sequence).

These clones are 2-2 circles using canonical splice sites.

*YOS1 2G19#1:*

CCACGAAGTCCAAGGTTGTTCAATTGATTGGCGCCGTACAGACATTACTGAGGATTGGCCTTGGAAGATCAAATGATGAGACACCTGTTTTTGGTCA-

*YOS1 2G19#3:*

CCACGAAGTCCAAGGTTGTTCAATTGATTGGCGCCGTACAGACATTACTGAGGATTGGCCTTGGAAGATCAAATGATGAGACACCTGTTTTTGGTCAGGGTCAAAATACCACGAAGTCCAAGGTTGTTCAATTGATTGGCGCCGTACAGACATTACTGAGGATTGGCCTTGGAAGATCAAATGATGAGACACCTGTTTTTGGTCAGGGTCAAAATACCACGAAGTCCAAGGTTGTTCAATTGATTGGCGCCGTACAGACATTACTGAGGATTGGCCTTGGAAGATCAAATGATGAGACACCTGTTTTTGGTCAGGATCAAAATACCACGAAGTCCAAGGTTGTTCAATTGATTGGCGCCGTACAGACATAAATGAGGATTGGCCTTGGAAGATCAAATGATGAGACACCTGTTTTTGGTCAGG

*YOS1 2E19#5:*

CCACGAAGTCCAAGGTTGTTCAATTGATTGGCGCCGTACAGACATTACTGAGGATTGGCCTTGGAAGATCAAATGATGAGACACCTGTTTTTGGTCAGGATCAAAATACCACGAAGTCCAAGGTTGTTCAATTGATTGGCGCCGTACAGACATTACTGAGGATTGGCCTTGGAAGATCAAATGATGAGACACCTGTTTTTGGTCAGGATCAAAATACCACGAAGTCCAAGGTTGTTCAATTGATTGGCGCCGTACAGACATTACTGAGGATTGGCCTTGGAAGATCAAATGATGAGACACCTGTTTTTGGTCA--

*YOS1 2E19#3:*

CCACGAAGTCCAAGGTTGTTCAATTGATTGGCGCCGTACAGACATTACTGAGGATTGGCCTTGGAAGATCAAATGATGAGACACCTGTTTTTGGTCAGGATCAAAATACCACGAAGTCCAAGGTTGTTCAATTGATTGGCGCCGTACAGACATTACTGAGGATTGGCCTTGGAAGATCAAATGATGAGACACCTGTTTTTGGTC-G-

*YOS1 2E19#1:*

CCACGAAGTCCAAGGTTGTTCAATTGATTGGCGCCGTACAGACATTACTGAGGATTGGCCTTGGAAGATCAAATGATGAGACACCTGTTTTTGGTCAGGAGACACCTGTTTTTGGTCAGGGTCAAAATACCACGAAGTCCAAGGTTGTTCAATTGATTGGCGCCGTACAGACATTACTGAGGATTGGCCTTGGAAGATCAAATGATGAGACACCTGTTTTTGGTCAGGATCAAAATACCACGAAGTCCAAGGTTGTTCAATTGATTGGCGCCGTACAGACATTACTGAGGATTGGCCTTGGAAGATCAAATGATGAGACACCTGTTTTTGGTCA-

These clones are consistent with being 2-1 circles, splicing to two different cryptic splice sites in exon 1. Exon 1 sequences are indicated in lowercase red.

*YOS1 1E19#4:*

CCACGAAGTCCAAGGTTGTTCAATTGATTGGCGCCGTACAGACATTACTGAGGAactattttacgtcattttattattgattaatgccgttgcagtactgagcgaagaaaggttcttaagaagaaTTGGCCTTGGAAGATCAAATGATGAGACACCTGTTTTTGGTCAGGATCAAAATACCACGAAGTCCAAGGTTGTTCAATTGATTGGCGCCGTACAGACATTACTGAGGATTGGCCTTGGAAGATCAAATGATGAGACACCTGTTTTTGGTC-G-

*YOS1 1E19#1:*

CCACGAAGTCCAAGGTTGTTCAATTGATTGGCGCCGTACAGACATTACTGAGGAactattttacgtcattttattattgattaatgccgttgcagtactgagcgaagaaaggttcttaagaagaaTTGGCCTTGGAAGATCAAATGATGAGACACCTGTTTTTGGTCAGGGTCAAAATACCACGAAGTCCAAGGTTGTTCAATTGATTGGCGCCGTACAGACATTACTGAGGAactattttacgtcattttattattgattaatgccgttgcagtactgagcgaagaaaggttcttaagaagaaTTGGCCTTGGAAGATCAAATGATGAGACACCTGTTTTTGGTCAGGGTCAAAATACCACGAAGTCCAAGGTTGTTCAATTGATTGGCGCCGTACAGACATTACTGAGGAgtagactattttacgtcattttattatagattaatgccgttgcagtactgagcgaagaaaggttcttaagaagaaTTGGCCTTGGAAGATCAAATGATGAGACACCTGTTTTTGGTCA-

*YOS1 1E19#5:*

CCACGAAGTCCAAGGTTGTTCAATTGATTGGCGCCGTACAGACATTACTGAGGAactattttacgtcattttattattgattaatgccgttgcagtactgagcgaagaaaggttcttaagaagaaTTGGCCTTGGAAGGTCAAATGATGAGACACCTGTTTTTGGTCAGG
