# Supplementary material for: Correction: Circular RNA Is Expressed across the Eukaryotic Tree of Life
Source: PLoS One. 2014 Apr 8;9(4):e95116. doi: 10.1371/journal.pone.0095116 (PMC3979859; doi:10.1371/journal.pone.0095116)
Supplement: File S1 — Originally published, uncorrected article. (PDF) [file pone.0095116.s001.pdf]

Circular RNA Is Expressed across the Eukaryotic Tree of Life

Peter L. Wang[1,2], Yun Bao[3,4,5], Muh-Ching Yee[3,4,5], Steven P. Barrett[1], Gregory J. Hogan[1], Mari N. Olsen[1,6¤], José R. Dinneny[3,4,5], Patrick O. Brown[1,6], Julia Salzman[1,2]*

1 Department of Biochemistry, Stanford University School of Medicine, Stanford, California, United States of America, 2 Stanford Cancer Institute, Stanford University School of Medicine, Stanford, California, United States of America, 3 Department of Plant Biology, Carnegie Institution for Science, Stanford, California, United States of America, 4 Temasek Lifesciences Laboratory, National University of Singapore, Singapore, Singapore, 5 Department of Biological Sciences, National University of Singapore, Singapore, Singapore, 6 Howard Hughes Medical Institute, Stanford University School of Medicine, Stanford, California, United States of America

Abstract

An unexpectedly large fraction of genes in metazoans (human, mouse, zebrafish, worm, fruit fly) express high levels of circularized RNAs containing canonical exons. Here we report that circular RNA isoforms are found in diverse species whose most recent common ancestor existed more than one billion years ago: fungi (*Schizosaccharomyces pombe and Saccharomyces cerevisiae*), a plant (*Arabidopsis thaliana*), and protists (*Plasmodium falciparum* and *Dictyostelium discoideum*). For all species studied to date, including those in this report, only a small fraction of the theoretically possible circular RNA isoforms from a given gene are actually observed. Unlike metazoans, *Arabidopsis*, *D. discoideum, P. falciparum, S. cerevisiae,* and *S. pombe* have very short introns (~100 nucleotides or shorter), yet they still produce circular RNAs. A minority of genes in *S. pombe* and *P. falciparum* have documented examples of canonical alternative splicing, making it unlikely that all circular RNAs are by-products of alternative splicing or 'piggyback' on signals used in alternative RNA processing. In *S. pombe*, the relative abundance of circular to linear transcript isoforms changed in a gene-specific pattern during nitrogen starvation. Circular RNA may be an ancient, conserved feature of eukaryotic gene expression programs.

Citation: Wang PL, Bao Y, Yee M-C, Barrett SP, Hogan GJ, et al. (2014) Circular RNA Is Expressed across the Eukaryotic Tree of Life. PLoS ONE 9(3): e90859. doi:10.1371/journal.pone.0090859

Editor: Thomas Preiss, The John Curtin School of Medical Research, Australia

Received November 22, 2013; **Accepted** February 5, 2014; **Published** March 7, 2014

Copyright: © 2014 Wang et al. This is an open-access article distributed under the terms of the Creative Commons Attribution License, which permits unrestricted use, distribution, and reproduction in any medium, provided the original author and source are credited.

Funding: This work was supported mainly by the Howard Hughes Medical Institute. POB is an investigator for the Howard Hughes Medical Institute. PLW was supported by a grant from the Canary Foundation. JS was supported by NIH NCI 1K99CA168987-01. The funders had no role in study design, data collection and analysis, decision to publish, or preparation of the manuscript.

Competing Interests: The authors have declared that no competing interests exist.

* E-mail: julia.salzman@stanford.edu

¤ Current address: University of California Santa Cruz and Howard Hughes Medical Institute, Santa Cruz, California, United States of America

Background

Until recently, circular RNA isoforms have largely gone unnoticed, with some notable exceptions [1–8]. Yet RNA circles are expressed from a large fraction of human, mouse and *Drosophila* genes and constitute the major RNA isoform from hundreds of these genes [9,10]. Circular RNA molecules contain exons from coding or noncoding transcripts spliced in an unconventional order – a downstream splice donor spliced to an upstream splice acceptor site. Since our initial report of widespread RNA circles in humans and mouse, abundant circular RNAs have been reported in other metazoans, including zebrafish, *C. elegans* and fruit flies; and confirmed by other groups in human and mouse cells [11,12].

Circular splicing of RNAs appears to be regulated: for many genes, the ratio of circular to linear transcripts and the relative abundance of differentially spliced circular isoforms is cell-type specific. Introns are retained in some circular RNAs, adding an additional layer of complexity to the circular RNA transcriptome [10]. In many human cell lines, including fetal fibroblasts (AG04450) and HeLa cells, the overall abundance of circular RNA molecules is equivalent to 1% of the abundance of poly(A) RNA [10].

Splicing is a ubiquitous feature of eukaryotic gene expression, and it is now thought that the "Last Common Eukaryotic Ancestor" (LECA) was relatively intron-rich and had complex spliceosome machinery and splicing signals [13,14]. Given that circular RNAs generated by splicing have been observed now in a range of metazoans, it seemed possible that they might also be found in even more disparate branches of the eukaryotic tree of life (see Figure 1). Genomic architectures vary considerably at these larger evolutionary distances, with many organisms having significantly shorter average introns than metazoans, and with varying prevalence of alternative splicing. The RNA regulatory toolkit also varies; some species lack microRNA pathways entirely, others have even more elaborate small RNA-mediated regulatory mechanisms such as gene silencing. The identification of circular RNAs in highly divergent species would raise intriguing questions about their evolutionary history, functions, and minimal genomic requirements for their biogenesis.

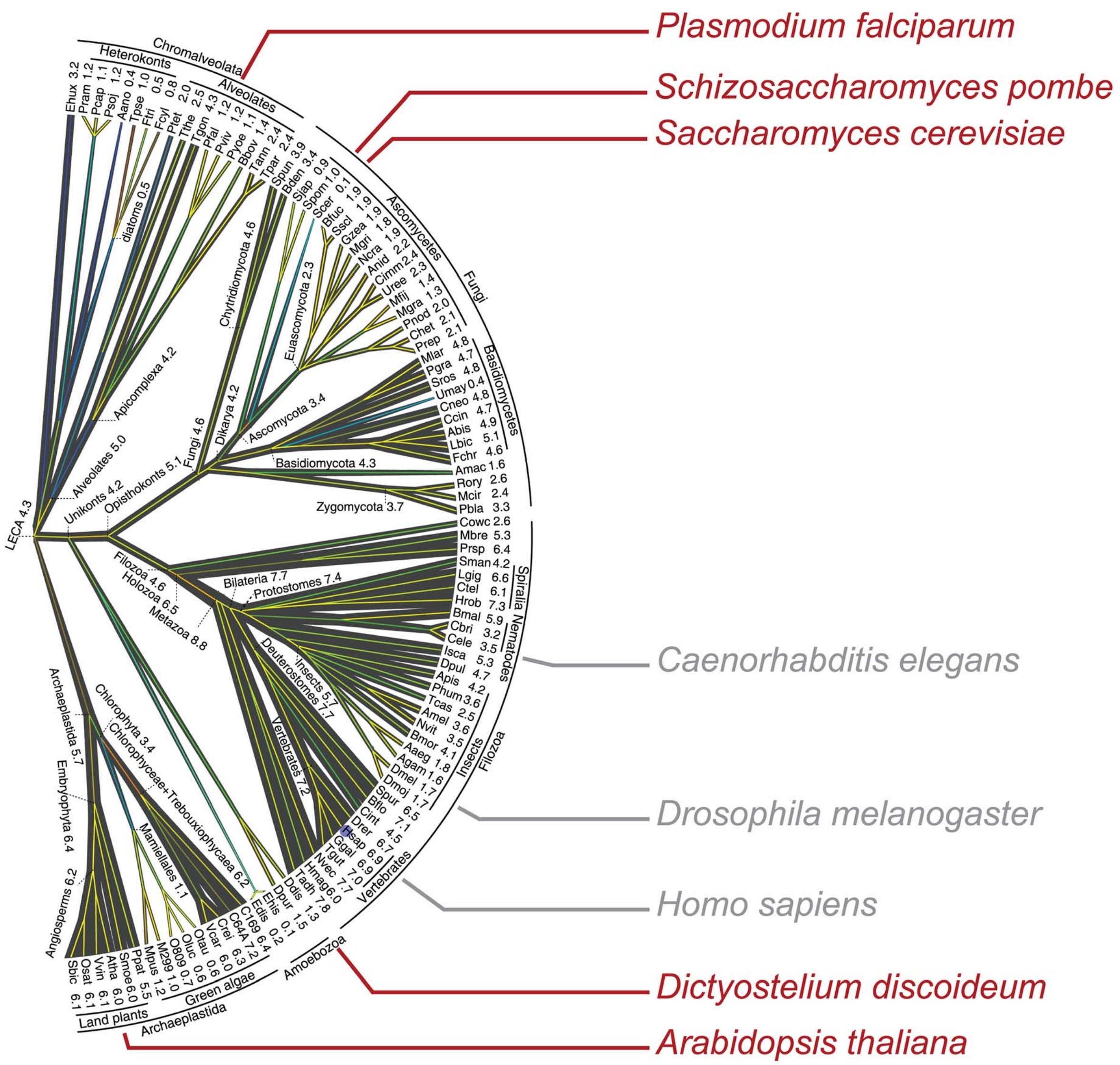

Figure 1. Eukaryotic Tree of Life. This shows the divergence between organisms studied in this report (in red) and metazoans where circular RNA expression has been previously reported. Adapted from Csuros *et al.* [14] under Creative Commons license CC0. According to [14], "Branch widths are proportional to intron density which is shown next to terminal taxa and some deep ancestors, in units of the introns count per 1 kb coding sequence".
doi:10.1371/journal.pone.0090859.g001

Results

Evidence from RNA-Seq data for circular RNA isoforms in diverse organisms

We mined RNA-Seq libraries from three species (shown in red in Figure 1) for evidence of circular RNA. The protocols differed in their methods for RNA selection and library preparation (see Methods). We focused on analyzing sequence data from libraries prepared with minimal selection for polyadenylated RNA, as the circular RNAs we previously detected lack poly(A) tails. However, we and others have detected evidence of "scrambled exons" attributable to circular RNA transcripts even in libraries prepared from poly(A)-selected RNA (e.g., [8,15,16]), and reasoned that even sequence datasets from poly(A)-selected RNA could provide evidence of circular RNA expression even if not for quantitative estimates of abundance. To this end, we performed a very basic bioinformatic analysis by aligning RNA-Seq reads to custom databases of exon-exon junctions, generated as previously described (see Methods) [9]. Reads mapping to junctions between exons in non-canonical order ("exon scrambling") were used to generate a list of putative circular isoforms in each organism. This sequence analysis provided evidence of multiple circular RNAs in the fungus *Schizosaccharomyces pombe*, the protist *Plasmodium falciparum* and the plant *Arabidopsis thaliana*, respectively. In addition, we prepared an RNase R treated RNA-Seq library from *Dictyostelium discoideum* and performed the same bioinformatic analysis described above.

Predicted circular RNAs amplify with circle-specific primers and are exonuclease-resistant

We chose a handful of circular RNA candidates from each of the three species for additional experimental validation and analysis. Most were selected from candidates that had the highest apparent abundance based on the RNA-Seq data, but some were

selected to represent apparently lower-abundance circular species or for other biological reasons. Our methodology for testing circular RNA candidates is illustrated in Figure 2a for one of the genes. cDNA derived from circular RNAs, but not canonically spliced linear RNA from the same gene, would be expected to PCR-amplify with primers that are "outward-facing" with respect to the canonical linear RNA (circle-specific), that is, where the forward primer is located 3′ of the reverse primer when aligned to the genomic sequence. As a control, we also designed primer-pairs that should amplify the linear RNA outside of the region implicated in the circular isoform (linear-specific).

PCR products from circle-specific primers were resolved by agarose gel electrophoresis. In *S. pombe*, four of the primer pairs we tested amplified a product of the size expected for the predicted circle. For some of the candidates from *Arabidopsis* and *P. falciparum*, in addition to a product of the expected size there were also products of other sizes (see Figure 2a for an example). All of the circular-RNA candidates we tested in *S. pombe* and *Arabidopsis* (four each) gave circle-specific PCR products. The success rate in *P. falciparum* was lower; only two candidates gave a circle-specific PCR product, six gave no product (all eight genes gave a linear-specific product). While for *S. pombe* and *Arabidopsis* we were able to test RNA from conditions identical to those in the datasets used for discovery, we could not be sure of this for *P. falciparum* because the dataset had little sample annotation.

The circle-specific PCR products were sequenced, either directly when a single band was the product, or after cloning when multiple bands were observed (Table 1). (We were unable to clone one of the *Arabidopsis* circular-RNA candidates, so it was not further pursued). In all the other cases, the sequence results confirmed the expected circle-junction sequence (see Text S1). Where the PCR showed more than one band, sequencing of

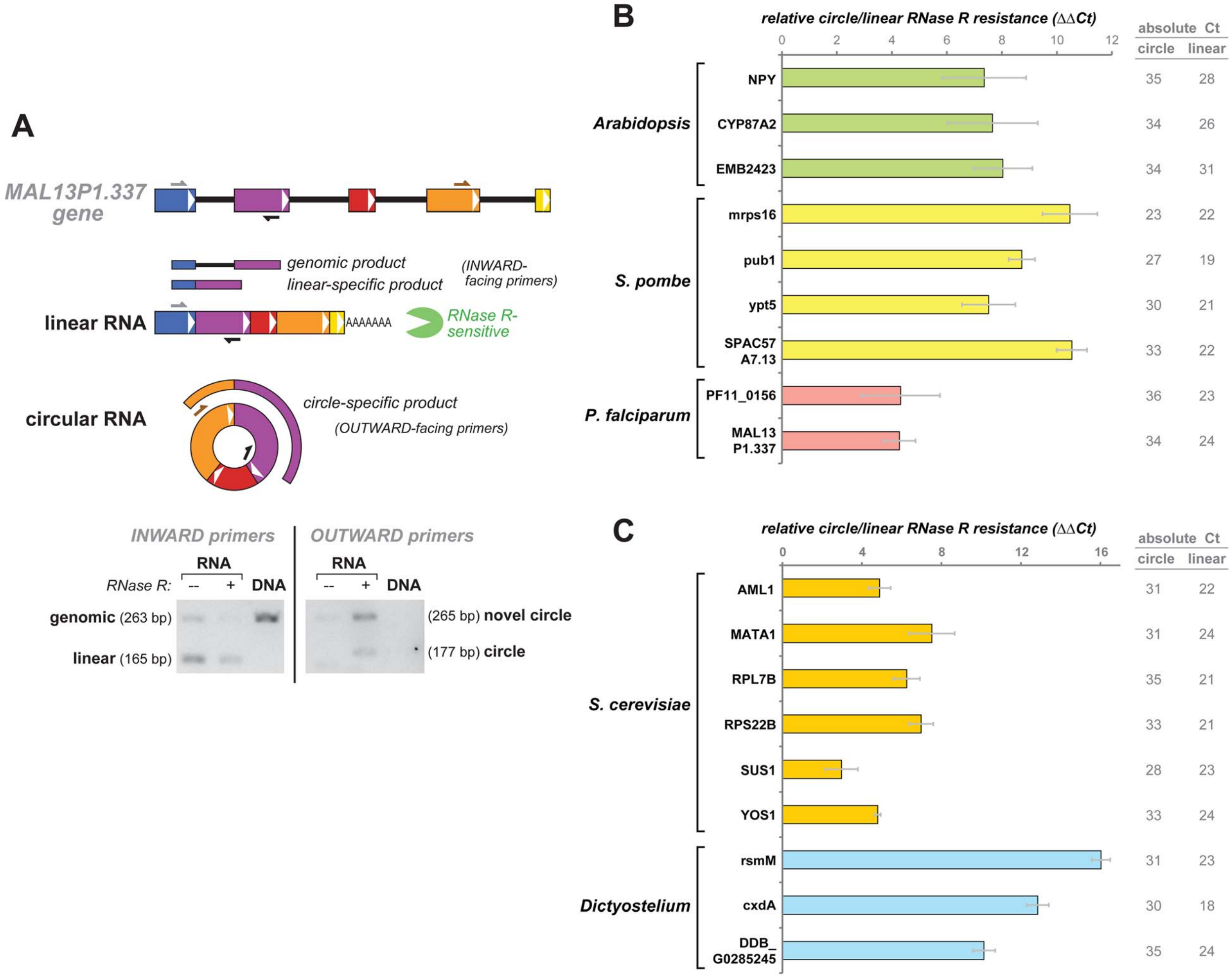

Figure 2. Circle-specific PCR and relative RNase R resistance. a) An example of circular and linear isoforms, in this case for the *P. falciparum* gene *MAL13P1.337*, and circle- and linear-specific PCR design. PCR is performed on cDNA from total RNA that was mock-treated or RNase R-treated, or on *P. falciparum* genomic DNA. Circle-specific PCR amplifies from RNA but not genomic DNA; it amplifies the candidate junction (177 bp band) but also an unexpected band corresponding to a 4-1 circle. b) Quantitation of RNase R resistance. Plotted here is the *relative* RNase R resistance of a circular isoform compared to its counterpart linear isoform ($\Delta\Delta$Ct): RNase R resistance(circle) – RNase R resistance(linear); gray bars are standard errors. RNase R resistance (the $\log_2$ fold-change in RNA isoform abundance with RNase R treatment) was measured by quantitative RT-PCR and taken as ΔCt = Ct(mock – treatment) – Ct(RNase R – treatment). RNase R resistance values for circular and linear isoforms are separately shown in Figure S1. All linear isoforms were sensitive to RNase R, showing a greater than 32-fold drop in abundance after RNase R treatment (ΔCt <-5). Circular isoforms show no significant decrease in abundance with RNase R treatment, and in many cases the signal actually increases (see main text). The absolute Ct for mock-treated RNA is also given, as an indicator of the comparative abundance of circular and linear isoforms. For *S. pombe*, data shown here is for exponential growth. c) RNase R resistance of genes in two additional organisms, *Dictyostelium* and *S. cerevisiae*. Format is similar to b).
doi:10.1371/journal.pone.0090859.g002

Table 1. List of genes for which circles were validated including organism, number of exons per gene, circle exon-exon junctions observed, and gene descriptors.

Gene	locus tag	exons	observed circles	''Gene description'', other notes
Schizosaccharomyces pombe				
mrps16	*SPBC354.06*	3	2-2	''mitochondrial ribosomal protein subunit S16 (predicted)''
pub1	*SPAC11G7.02*	4	3-3	''HECT-type ubiquitin -protein ligase E3 Pub1''
SPAC57A7.13	*SPAC57A7.13*	4	2-2	''RNA-binding protein, involved in splicing (predicted)''
ypt5	*SPAC6F6.15*	9	7-6	''GTPase Ypt5'', rab5 homolog, endosome fusion regulator
Arabidopsis thaliana				
CYP87A2	*AT1G12740*	9	5-4, 5a-5a, 5a-4, 6a-4	''cytochrome P450, family 87, subfamily A, polypeptide 2''
EMB2423	*AT3G48470*	13	8a-6	''protein embryo defective 2423'', embryo-defective phenotype, homology to telomere-length regulation protein
NPY4	*AT2G23050*	4	3-3	''BTB/POZ domain-containing protein NPY4'', involved in gravitropic response [40]
Plasmodium falciparum				
MAL13P1.337		5	4-2, 4-1b, 2-2, 2-1a	''Skp1 family protein, putative'', SCF ubiquitin ligase subunit
PF11_0156		9	4-4, 4-3	''Ser/Thr protein kinase''
Dictyostelium discoideum				
rsmM	*DDB_G0292300*	7	5-4	''small GTPase''
cxdA	*DDB_G0281393*	4	2-2	''cytochrome c oxidase subunit IV''
DDB_ G0285245	*DDB_G0285245*	4	2-2	''hypothetical protein''
Saccharomyces cerevisiae				
AML1	*YGR001C*	3	2-2	''hypothetical protein''
MATA1 (HMRA1)	*YCR097W*	3	2-2	homeobox corepressor involved in mating type
RPL7B	*YPL198W*	3	2-2	''ribosomal 60S subunit protein L7B''
RPS22B	*YLR367W*	3	2-2	''ibosomal 40S subunit protein S22B''
SUS1	*YBR111W-A*	3	2-2	involved in mRNA export and histone H2B deubiquitination
TAD3	*YLR316C*	3	2-1a, 2-1b	subunit of tRNA-specific adenosine-34 deaminase
YOS1	*YER074W-A*	3	2-2, 2-1a, 2-1b	integral membrane protein required for ER to Golgi transport

For exon-exon junctions, exon numbers suffixed by a letter indicate usage of a different 5′ or 3′ splice site than in the genome annotation.
doi:10.1371/journal.pone.0090859.t001

individual clones showed that these were also consistent with circular RNA, either novel isoforms involving additional exons or repeats due to rolling-circle reverse transcription.

Unannotated genomic duplications or rearrangements could also give rise to transcripts with exons spliced in a non-canonical order consistent with circular RNA. Because the genomes of *P. falciparum* and *Arabidopsis* are not comprehensively annotated, we tested each set of circle-specific primers with genomic DNA as a template in these organisms. We found no evidence of genomic rearrangements: inward-facing (canonical linear-specific) primers amplified DNA with products of expected size, while outward facing primers failed to give a specific product (see Figure 2a for example).

Resistance to exonuclease digestion is a distinctive property of circular RNAs, owing to their lack of 5′ or 3′ ends. We therefore expect circular RNAs to be much more resistant than conventional linear RNAs to RNase R, a RNA-specific, highly processive 3′ to 5′ exonuclease that digests essentially all linear RNAs with at least seven free unpaired nucleotides at the 3′ end (and has activity even on some RNAs with shorter free 3′ tails) [17]. We therefore measured the fractional recovery of each RNA isoform after RNase R or mock treatment, respectively, by quantitative RT-PCR with circle-specific and linear-specific primers. Circular

isoforms were much more resistant to RNase R than their corresponding linear isoforms (Figures 2b and 2c; Figure S1). All linear isoforms decreased in abundance by 6-fold or more with RNase R treatment, while none of circular isoforms decreased significantly; indeed many increased in apparent abundance after RNase R treatment (reflecting increased reverse transcription efficiency due to the overall decrease in RNA input). This pattern is exemplified in Figure 2a, where following separation by agarose gel electrophoresis, both circular isoform bands were stronger in the RNase R-treated lane than in the mock-treated lane.

We also report in Figure 2b and 2c the absolute Ct values for both circle-specific and linear-specific qRT-PCRs. The higher circle Cts indicate that, in all but one case (*mrps16* in *S. pombe*) studied here, the circular isoform was present at significantly lower abundance than its corresponding linear isoform.

Circular RNA is expressed in the *Arabidopsis thaliana* root

We analyzed sequence data obtained from RNA extracted from the root of *Arabidopsis thaliana* and depleted of ribosomal RNA, followed by cDNA synthesis using a mix of oligo(dT) and random hexamers as primers (YB, MCY, JRD *et al.*, manuscript in preparation). By the criteria described in the preceding section, we validated three genes with evidence of circular RNA expression: a circle consisting of only exon 3 of *NPY4*, a gene implicated in gravitropic response; an exon 6-7-8 circle in *EMB2423*, a telomere-length regulation protein homolog; and an exon 4–5 circle in *CYP87A2*, a cytochrome P450 enzyme. For *CYP87A2*, we also identified circles composed of exon 5 only and exons 4-5-6; in addition, some circular isoforms used alternative 5′ and 3′ splice sites; the latter phenomenon was seen in *EMB2423* as well (see Table 1 and Text S1).

It has been proposed that some circular RNA isoforms could be produced secondarily by splicing from a lariat excised during exon-skipping, as it was observed that the exons present in some circular isoforms of a rat cytochrome P-450 gene were precisely those lacking in alternative mRNA transcripts arising from exon-skipping [18]. This pattern has not proven to be the general rule for circular isoforms [10]. For all three genes, we searched for exon-skipping in canonical alternatively spliced transcripts using the same *Arabidopsis* dataset and exon-exon junction database used for circle discovery (see Methods). For *CYP87A2*, encoding a cytochrome P-450, we did indeed detect spliced transcripts that skipped exons 4 and 5 (i.e. with sequence reads representing an exon 3 - exon 6 junction), precisely the exons present in the predominant circular isoform identified for this gene. For the additional circular isoforms of this gene's transcripts (comprising exon 5, or exons 4-5-6) we found no evidence of the complementary exon-skipping linear transcripts. Nor did the other two *Arabidopsis* genes we chose for validation show circular RNA exon-skipping reciprocity: we found no evidence for exon-skipping RNA isoforms of *NPY4*, and although one transcript isoform of *EMB2423* skipped exon 2, this skipped exon had no obvious relevance to the observed circular isoforms, which comprised exons 6, 7 and 8.

Plasmodium falciparum expresses low abundance circular RNAs

In a simple bioinformatic search for circular RNAs using 60 nt RNA-Seq libraries from *P. falciparum*, we found evidence for several circular RNA species. All were represented at low levels in the RNA-Seq data. The abundance of the candidate circular RNA species relative to their linear counterparts varied among libraries (which we presume represented different life-cycle stages). All required many PCR cycles for detectable amplification.

Based on our analysis of RNA-Seq data, we predicted a circle composed of exons 3 and 4 of the gene *PF11_0156*, a putative serine/threonine protein kinase. This predicted circular isoform was confirmed using outward facing primers in exon 4; in addition, we also detected a circle composed only of exon 4 (see Table 1 and Text S1). We did not find evidence for canonical exon skipping in *PF11_0156*.

We also predicted that *P. falciparum* expressed three circular isoforms of *MAL13P1.337*, an SCF ubiquitin ligase subunit, depicted as an exemplary gene in Figure 2a. The exons contained in these predicted circles were: a) 2; b) 2-3; and c) 2-3-4. RNase R treatment and sequencing of cloned PCR products confirmed isoforms a) and c), as well as two other circular isoforms that would not have been detected using our algorithm as they use splice sites not annotated in the current version of the genome (see Table 1 and Text S1). The findings here are consistent with the production of multiple distinct circular RNA isoforms by alternative splicing from the *MAL13P1.337* gene, a phenomenon also observed for circles from several human genes [10]. The only evidence we found for canonical alternative splicing of *MAL13P1.337* transcripts was for a very low level of skipping of exon 4.

Dictyostelium discoideum expresses circular RNA

Although we identified putative circle junctions in public RNA-Seq data from the social amoeba *Dictyostelium discoideum*, we had difficulty validating candidates by PCR in our own *Dictyostelium* RNA. To pursue the presence or absence of circular RNA in *Dictyostelium*, we prepared a low coverage RNA-Seq library from RNase R treated RNA isolated during the vegetative growth phase of *Dictyostelium*. This library suffered from a high fraction of contaminating DNA and hence was not a comprehensive survey of circular RNA in *Dictyostelium*; it was also sequenced to a shallow depth (296,534 paired-end 150 nt reads, multiplexed with several other unrelated libraries). We identified 3 putative circular RNAs which were all subsequently verified by qRT-PCR as being RNase R-resistant (Figures 2c and S1b) and having the expected noncanonical exon junctions by Sanger sequencing (Text S1): an exon 5-4 circle from the gene *rsmM*, a ras superfamily member small GTPase; an exon 3-2 circle from the gene *cox4*, cytochrome c oxidase subunit IV and an exon 2 circle from the gene *DDB0237733* of unknown function.

The genetically tractable model organism *S. pombe* expresses circular RNAs that change in abundance during nitrogen starvation.

Schizosaccharomyces pombe is a model eukaryotic organism, with a particularly well-developed set of genetic and cell-biological resources and experimental methods for systematic studies of the molecular architecture of physiological and regulatory systems. We searched for circular RNA expression in the data of Marguerat *et al.* [19], who sequenced *S. pombe* total RNA from two conditions: 1) exponential growth in complete minimal medium, and 2) after 24 hours of nitrogen starvation, and performed careful quantitative analysis of this data. Upon nitrogen starvation, *S. pombe* cells divide twice (decreasing in size), and either mate then undergo meiosis, or else arrest in a stress-resistant quiescent state [20]. Although sequences from ribosomal RNAs comprised the vast majority of reads in these libraries, because they were sequenced very deeply each library contains more than 5 million reads mappable to mRNA.

We identified sequences indicative of circular RNAs from 42 genes - roughly 1% of the 5110 annotated protein coding genes in *S. pombe* and 3% of the 1374 genes with 2 or more introns. The

majority of these candidate circles were represented by single reads (see Table S1). We selected four genes for further validation: *mrps16* (a mitochondrial ribosomal protein), *pub1* (a ubiquitin ligase), *ypt5* (a Rab GTPase), and *SPAC57A7.13* (a putative splicing-related RNA-binding protein). All four gave circle-specific RT-PCR products of the expected size and sequence, and all were resistant to RNase R treatment (see Table 1, Figures 2b and S1a, and Text S1). The exon structure of these genes is shown in Figure 3; the overall sizes of these *S. pombe* genes, as well as their intron sizes, is considerably smaller than most human genes that produce circles, two examples of which are shown for comparison.

There were about 10-fold more circle junction reads identified in the RNA from cells after 24 hr of nitrogen starvation compared to RNA from exponentially growing cells. From the RNA-Seq data, we can estimate the relative ratio of total circular RNA molecules to total linear mRNA molecules in each condition (see Methods). By multiplying by the number of mRNAs per cell as estimated by Marguerat *et al.* (~41,000 in exponential growth, ~7300 in nitrogen starvation), we obtain a rough estimate of ~1.4 molecules of circular RNA per cell in exponential growth, and ~4.2 circles per cell in nitrogen starvation. The true circle abundance may be higher; circles may be systematically under-represented in these data-sets as the majority of circles are smaller than the approximately 200 nt fragment size targeted for library construction (see Table S1). In any case, mRNA molecules per cell decrease dramatically during nitrogen starvation while their circular RNA counterparts do not; thus the ratio of total circular RNA molecules to total mRNA molecules goes up by an order of magnitude during nitrogen starvation.

We used quantitative RT-PCR to examine the relative abundance changes of individual RNA species during a time-course of nitrogen starvation of *S. pombe*. As documented by Marguerat *et al.*, cell size and total RNA content per cell decrease dramatically during nitrogen starvation; the majority of linear mRNAs decrease their copy number per cell [19], and that is true for the linear isoforms of *mrps16*, *pub1*, *ypt5*, and *SPAC57A7.13* (Figure 4). The circular isoform of *ypt5* decreases in parallel with the linear isoform; the circular isoform of *SPAC57A7.13* decreases faster than its corresponding linear isoform. By contrast, the *mrps16* and *pub1* circles show comparatively steady levels during nitrogen starvation. Circular isoforms might be expected to be more resistant to degradation compared to their linear counterparts due to the lack of ends that could be attacked by exonucleases as well as their generally smaller size (and therefore smaller target size for endonucleases); data from human cells is consistent with this idea [11]. However, the differences in temporal patterns between circular isoforms seen here suggest that, just as for linear mRNAs, there may be dynamic regulation of the balance between degradation and production of circular RNAs, in response to environmental conditions.

The extensively studied model organism *S. cerevisiae* expresses circular RNAs

Although RNA splicing has been extensively studied in the yeast *S. cerevisiae,* very few of its genes contain introns; of these only ten have more than one intron (all ten have exactly two introns [21]), a presumed requirement for spliceosomal RNA circle formation. We tested nine of these ten genes for evidence of circle formation by RT-PCR with outward facing primers located in the middle (second) exon of each gene (this strategy was not possible for one gene, *DYN2*, whose middle exon is only 23 bp). We found evidence for circular transcript isoforms from the majority of these two-intron genes: products of the size expected from the predicted circular RNA isoforms, amplified by PCR using the outward-facing primers, resistant to RNAseR treatment of the template RNA, with exon junction sequences consistent with circular splicing (see Text S1). For six of the genes the circular-splice junction was at the precise boundaries of exon 2; qRT-PCR data for these are shown in Figures 2c and S1b. An additional putative circular transcript, *TAD3*, also showed RNase R resistance; Sanger

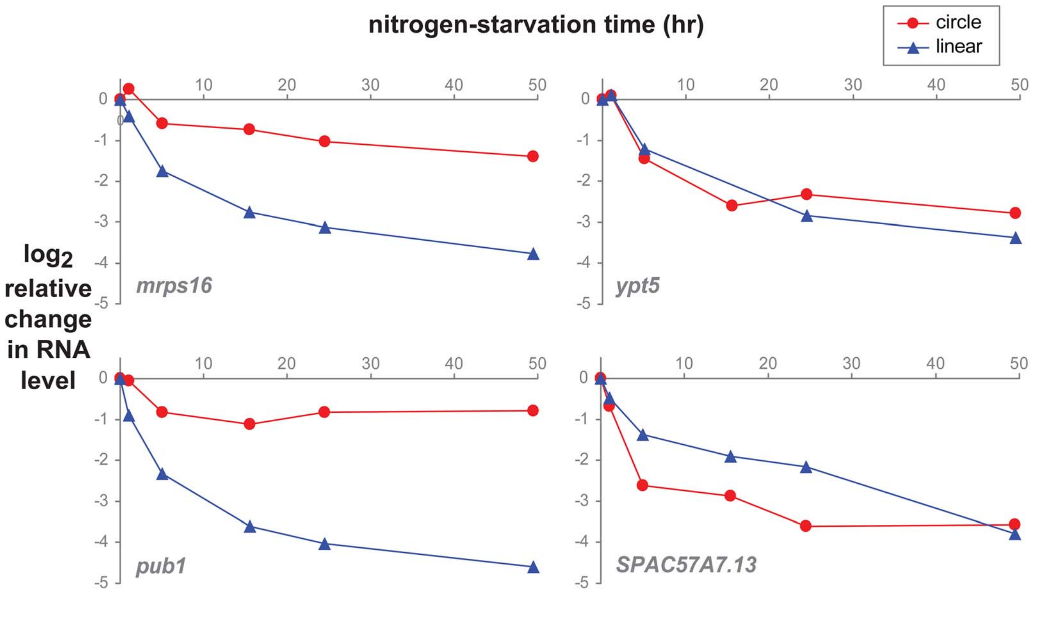

Figure 3. *S. pombe* and human genes producing circular RNAs. The four *S. pombe* genes for which we validated circular isoforms are shown schematically. Exons are boxes, with untranslated regions in light green and coding regions in darker green, introns are indicated by a black line; the total size of the transcribed region is given in parentheses. The exons present in circular RNA are indicated by red boxes and their sizes indicated. For comparison, two human genes that produce circular isoforms are similarly presented, though at a very different scale since they are considerably larger and contain much larger introns.

doi:10.1371/journal.pone.0090859.g003

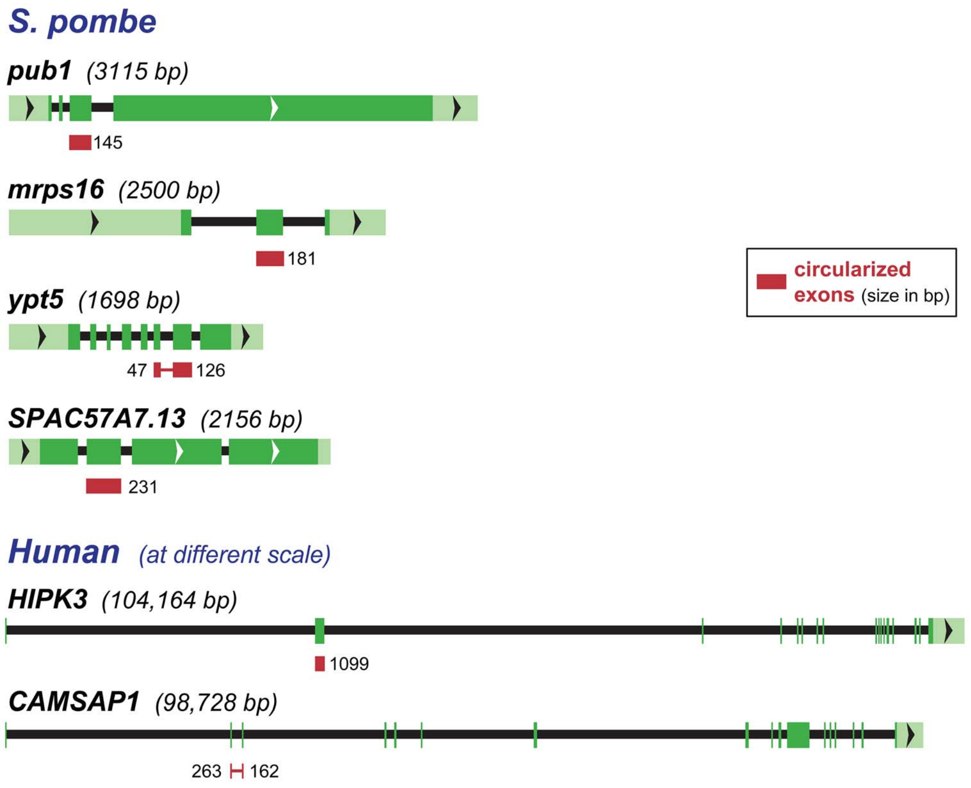

Figure 4. *S. pombe* circular and linear RNA changes during nitrogen starvation. *S. pombe* cultures were grown in complete minimal media to exponential phase. Time zero marks the switch to media lacking a nitrogen source. Relative RNA abundance (log_2 fold-change) of circular and linear isoforms was determined by quantitative RT-PCR (an equal mass of RNA for each timepoint was used as input), adjusted to a per cell basis, and expressed relative to time zero; the plotted value is Ct(time-zero) − Ct(time *n*) − log_2(RNA per cell at time *n*/RNA per cell at time-zero).
doi:10.1371/journal.pone.0090859.g004

sequencing of the RT-PCR products showed that the circles resulted from splicing the canonical exon 2 splice donor to cryptic splice-acceptor sites in exon 1 (see Text S1). Some of the sequences representing putative circular splice junctions in *YOS1* transcripts also fit a model of splicing the canonical exon 2 splice donor to a cryptic splice acceptor in exon 1, while others joined the canonical exon 2 splice donor and acceptor sites. Additional work, taking advantage of the experimental tractability of *Saccharomyces*, will be required to understand the biological activities of these circular RNAs.

Discussion

We have found evidence for circular RNA throughout the eukaryotic tree of life. Previous studies have reported circular RNA isoforms in mammals, fish, worms, and insects. In this study, we found clear evidence for RNA circles in two fungi (*Saccharomyces cerevisiae* and *Schizosaccharomyces pombe*), two protists (*Plasmodium falciparum* and *Dictyostelium discoideum*) and a plant (*Arabidopsis thaliana*). In addition to the data presented here, we have also preliminary bioinformatic evidence of circular RNA in several other organisms: the protist *Plasmodium yoelii*; the alga *Chlamydmonas reinhardtii*; and the filamentous fungus *Neurospora crassa* (one candidate validated by PCR, *NCU01564*). The occurrence of circular RNA with similar structural features in eukaryotes that diverged more than 1 billion years ago suggests that this aspect of gene expression is either deeply conserved, or the result of repeated convergent evolution.

Genome structures of the organisms described here differ greatly from those of metazoans in which circular RNA has been previously reported. Differences in intron structures are particularly interesting because most circular RNAs reported to date, including those reported here, have structures strongly suggesting that they are produced by mechanism similar or identical to canonical splicing, acting on canonical splice junctions in a non-canonical order. Previous studies of circular RNA expression have also suggested a possible relationship between features of the flanking introns and circular RNA production.

We previously noted a statistical enrichment for longer introns in genes from which circular RNA was transcribed and in the length of introns flanking exons participating as the donor or acceptor in a non-canonical splice junction generating the circular RNA, a finding also reported in a second study [9,11]. In a more comprehensive study of circular RNA expression, we found a less significant relationship between intron length flanking circularized exons, and that within a gene, flanking intron length was not explanatory of circular RNA biogenesis [10]. In a particular and perhaps exceptional case, circular RNA expression in the mouse *Sry* gene has been shown to require inverted nucleotide repeats in introns flanking the single exon of *Sry* [22].

An illustration of the differences in gene structure among these diverse species is shown in Figure 3, which depicts the four *S. pombe* circle-producing genes studied here, and two human circle-producing genes for comparison. The average intron length in humans is 5.4 kb; some human introns are hundreds of kilobases in length. Introns in *S. cerevisiae, S.pombe, Dictyostelium, P. falciparum* and *Arabidopsis* are miniscule by comparison, averaging 148, 82, 142,

134 and 158 nt, respectively, with standard deviations of the same order of magnitude [23]. Moreover, the gene *MATA1* in *S. cerevisiae* has among the shortest introns in the genome: both introns are less than 60 nt. As demonstrated by these organisms, long introns are clearly not required for circular RNA production.

Sequence features that specify splice sites, including the polypyrimidine tract, branch site and 5′ splice sites, as well as the splicing factors that mediate splicing reactions in these organisms have diverged in the more than 1 billion years separating them from metazoans. Splice sites in plants, fungi and protists are thought to specified by "intron definition" rather than "exon definition" used in human genes [24–28], although the true picture is likely more complex than this simple dichotomy [29]. Circular RNA production by "intron definition" would be particularly noteworthy, because it would have to involve, in effect, "defining" a discontinuous "intron".

The expression of numerous circular RNA isoforms in *S. pombe* and *P. falciparum* is also surprising because canonical alternative splicing is rare in these species: alternative splicing has been reported in *S. pombe*, but only a minority of genes (<1%) undergo exon skipping. Similarly, only a small minority – 254 (4.5%) – of genes in *P. falciparum* have been reported to undergo alternative splicing [30–32]. The occurrence of circular RNAs in species in which introns are small and alternative splicing rare argues against earlier suggestions that rare circular RNAs in some metazoans were byproducts of canonical alternative splicing, or arise from errors (mispairing of 3′ and 5′ splice sites) in complex splicing programs which rely on the spliceosome to identify small exons within long pre-mRNAs [3,18,33].

Recent reports on circular RNA in animals have proposed that they can function as microRNA sponges. CDR1 antisense transcript (CDR1as) is a circular RNA in mouse and human brain that contains more than 70 binding sites for the microRNA miR-7 and may suppress its activity. The mouse testis-specific circle of the *Sry* gene may likewise function to bind miR-138 [12,34]. However, there is little evidence that this is a general role for circular RNAs; indeed, *P. falciparum* lacks known siRNA or microRNA pathways [35] and *S. cerevisiae* has specifically lost these pathways [36].

During nitrogen starvation in *S. pombe*, the amount of linear mRNA per cell decreases dramatically [19]. Circular transcript isoforms from some genes remained relatively stable during nitrogen starvation; others decreased in a manner similar to their linear mRNA counterparts. This observation suggests the possibility that there are distinct control mechanisms for regulating the abundance of circular RNAs, and raises the possibility that the differential regulation may have yet to be discovered functional consequences.

Overall, the identification of circular RNAs in these diverse organisms challenges prevailing ideas about how circular RNAs are generated and what roles they may play. Their wide phylogenetic distribution multiplies the opportunities for investigation of these novel molecules. In particular, we predict that many circular RNAs may be regulated during development and environmental responses; exploring these in experimentally tractable organisms like *S. cerevisae*, *S. pombe* and *Arabidopsis* will complement the ongoing work on circular RNAs in humans and other metazoans.

Note:

While preparing our study for publication, we became aware of a study describing bioinformatic evidence of circular RNA isoforms in *S. pombe* [28]. The main aim of that study was to identify alternative splicing using an innovative approach involving physical purification and sequencing of intron lariats that accumulate in *S. pombe* mutant for the debranching enzyme Dbr1. In addition to abundant reads in lariats, they observed 11 exon-scramble reads but did not do any further validation. Two of these reads correspond to genes identified in this study, *mrps16* and *ypt5*. The *mrps16* junction is identical to the one we describe, while their *ypt5* isoform consisted of exon 4, intron 4, and exon 5 (distinct from the exon 8 – exon 7 isoform we describe). We did not identify their other exon-scramble genes in the analysis presented in this paper, nor did they identify exon-scramble reads for *pub1* and *SPAC57A7.13*. However, we subsequently analyzed the data from these authors [28] and found evidence of circle expression in *pub1* in addition to a total of 36 genes, in more than 50 reads (see Table S2). Notably, RNA-Seq data from this study was generated from *dbr1*-deleted cells that had been subjected to a variety of environmental stresses and pooled; in addition to their established limited ability to debranch lariats, *dbr1*-deleted cells show a severe growth defect [37].

Materials and Methods

Identification of circular RNA and datasets used

For each organism with RNA-Seq reads of length L, we constructed a custom sequence database of all possible intragenic exon-exon junctions as previously described [9]. Sequences corresponding to exon i – exon j junctions (with no constraint that $i<j$) were generated by taking $L-15$ bases at the 3′ end of exon i and $L-15$ bases at the 5′ end of exon j. In cases where the length of exon i or exon j was shorter than $L-15$, we padded out using tandem repeats of exon i – exon j (mimicking the expected product of "rolling-circle" reverse transcription of a circular RNA). In *P. falciparum* and *S. pombe*, all reads were mapped to this database without previous filtering, under bowtie conditions as below. Due to the extent of annotated alternative splicing in *Arabidopsis* and *Dictyostelium discoideum*, only reads that failed alignment to the annotated genome and transcriptome were mapped to the database.

The following RNA-Seq data, genome annotations, readlength, and bowtie alignment flags were used: -v 3 was used for reads exceeding 50nt and –m 1 was used for organisms which lack extensive annotated alternative splicing *(S. pombe and P. falciparum)*:

P. falciparum. Data was downloaded from the study: ERP001849. Annotations were downloaded from: http://www.broadinstitute.org/annotation/genome/plasmodium_falciparum_spp/MultiDownloads.html; read length = 60, bowtie flags: -v 3 -m 1.

S. pombe. Data was downloaded from the study: ERP001483. Annotations were downloaded from: ftp://ftp.ebi.ac.uk:21/pub/databases/pombase/pombe/GFF/; read length = 50; bowtie flags -v 1.

We also analyzed data from SRR927118, SRR927119 using the same annotations; reads were 43 nt and all alignments were performed with flags –trim5 3, reads failing alignment to the genome with flag –v 3 were aligned to the custom database as described above using the effective read length of $L=40$ and the flags –v 1 and –m 1.

Arabidopsis. Data will be reported in detail in another study (YB, MCY, JRD et al., manuscript in preparation). Annotations were downloaded from: ftp://ftp.arabidopsis.org/home/tair/. Only the first 10 annotated exons per transcript were used to generate the junctional database to conserve computational resources for the small minority of genes with >10 exons. Reads failing alignment to the genome and the transcriptome

(TAIR10_cdna_20101214_updated) under flags –v 3 were aligned to the junctional database; read length = 100; bowtie flags -v 3.

Dictyostelium discoideum. The genome and transcriptome were downloaded from ftp://ftp.ensemblgenomes.org/pub/protists/release-18/fasta/dictyostelium_discoideum/ and http://dictybase.org/db/cgi-bin/dictyBase/download/download.pl?area=blast_databases&ID=dicty_primary_cds.gz ([38]) respectively.

Reads failing alignment to the genome and the transcriptome were aligned to the junctional database; read length = 150; read length: 150; bowtie flags –trim5 5 –trim3 5 –v 3.

Estimation of circles per cell in *S. pombe*

RNA sequencing data and estimates of total mRNA copies per cell from Marguerat *et al.* [19] were used together to estimate the number of copies of circular RNA per cell. There were two replicates for each condition (exponential growth and nitrogen starvation); replicate values were averaged. For each condition, the rate R_{cir} of expression of each circular RNA transcript was taken as the number of reads spanning the diagnostic circular RNA junction; R_{cir} was summed across all genes to estimate the total expression of circular RNA. A rate R_{can} of expression of each canonical transcript was estimated by dividing the number of reads aligning to canonical exon-exon junctions in the gene and dividing by the number of such canonical junctions; R_{can} was summed over all transcripts to estimate the expression of multi-exon transcripts. S, the total copy number of single-exon genes, and M, the total copy number of multi-exon genes, were estimated from Supplemental Table 3 of Marguerat *et al.* The fraction of circular RNAs compared to linear mRNAs was estimated as: $\Sigma R_{cir}/\Sigma R_{can} \times M/(M+S)$. To estimate the number of circular RNAs per cell, this fraction was multiplied by the total number of mRNAs per cell (as estimated by Marguerat *et al.*: an average of 40,909 in exponential growth, and 7315 in nitrogen starvation).

Growth and nitrogen starvation and RNA isolation (*S. pombe)*

Wild-type *S. pombe* (972 h- strain) were grown in Edinburgh minimal medium (EMM) at 32°C with shaking to exponential phase (OD_{600} 0.25-0.3). The cells were centrifuged, washed with EMM-N (EMM lacking ammonium chloride), and resuspended in warm EMM-N to the original culture volume. The nitrogen-starved culture was continued at 32°C with shaking. Culture aliquots taken at various timepoints, cells counted, centrifuged, and cell pellets frozen at −80°C. RNA was purified from cell pellets by the hot-phenol method [39]. The RNA yield for 0, 5, 15, 24, and 49 hr was 2.8, 1.7, 0.83, 0.45, 0.36, and 0.29 pg/cell, respectively.

RNA isolation (*P. falciparum*)

P. falciparum total RNA was a kind gift of Joe DeRisi and Danny Ebert. Total RNA was harvested from a schizont-stage (~42–44 hours post-invasion) synchronized by 5% sorbitol treatment. Total RNA was extracted using Trizol (Invitrogen Corp., Carlsbad, CA, USA) followed by acid phenol chloroform extraction.

RNA isolation (*Arabidopsis*)

Total RNA from cultured *A.thaliana* root was extracted using RNeasy Plant Mini Kit (Qiagen) according to the manufacture's instructions.

RNase R treatment, cDNA synthesis, PCR and qPCR

1 microgram of total RNA was incubated with 5 U RNase R (Epicentre), 10 U murine Ribonuclease Inhibitor (New England Biolabs), in 1x RNase R buffer at 37°C for 30 min, then placed on ice. Mock reactions were the same but without addition of RNase R. cDNA synthesis followed without purification: 1 µl 1 mM EDTA, 1 µl dNTPs (10 mM each), and 1 µl random hexamer (100 µM) was added and the sample denatured at 65°C for 5 min, then placed on ice. 4 µl of 5× buffer (250 mM Tris pH 8, 125 mM KCl, 15 mM $MgCl_2$), 1 µl 0.1M DTT, 40 U murine Ribonuclease Inhibitor, and 1 µl reverse transcriptase (Protoscript II, NEB) was added, and the reaction incubated at 25°C for 10 min, 42°C for 50 min, 45°C for 5 min, 50°C for 5 min, 85°C for 5 min, 4°C hold.

cDNA reactions of mock and RNase R treated RNA were diluted with water and used as template for PCR. Standard PCR was done with Taq DNA polymerase (New England Biolabs); quantitative PCR was done with Power SYBR Green Mix (ABI/Life Technologies) on an ABI 7900HT. qPCR Ct values were calculated automatically by the manufacturer's software. A list of PCR primers is given in Text S1.

PCR products were either Sanger-sequenced directly using the amplification primers, or cloned into a TOPO vector (Invitrogen/Life Technologies) and sequenced with vector primers.

RNA-Seq in *Arabidopsis*

Total RNA was extracted from root tissue of seedlings (ecotype Col-0) using RNeasy Plant Mini Kit (Qiagen) according to the manufacture's instructions and quantified using the Qubit RNA Assay Kit (Invitrogen). Ribo-Zero Magnetic Kit (Plant Seed/Root) (Illumina) was used to remove rRNA from total RNA. The RNA was then fragmented using RNA Fragmentation Buffer (Kapa Biosystems). cDNA was prepared using SuperScript Double-Stranded cDNA Synthesis Kit (Invitrogen) using both random hexamer and oligo dT oligonucleotides. Library construction was performed using the KAPA HTP Library Preparation Kit (NEB) following the manufacture's instructions and amplified using custom-made barcoded adapters. Libraries were pooled together and sequenced on a HiSeq 2000 (Illumina) (Paired Reads, 2×101 bp plus Index Read).

RNA-Seq in Dictyostelium

Total RNA was extracted using TRIZOL from wildtype *Dictyostelium discoideum* cells (orfj) grown axenically in 1.5xHL+FM medium. RNA was treated with DNase I, digested twice with RNase R, combined with similarly treated *Neurospora crassa* RNA, and then processed to a barcoded paired-end Illumina sequencing library using 1/4th of a normal ScriptSeq reaction (Epicentre). This library was included as 1/15th of a pool of libraries for a single Illumina MiSeq 150 nt paired-end run.

Supporting Information

Figure S1 RNase R resistance of circular and linear isoforms. a) Quantitation of RNase R resistance. Plotted here is the RNase R resistance of each isoform (the $\log_2$ fold-change in RNA abundance with RNase R treatment), measured by quantitative RT-PCR and taken as ΔCt = Ct(mock-treatment) – Ct(RNase R-treatment). All linear isoforms were sensitive to RNase R, showing a greater than 32-fold drop in abundance after RNase R treatment ($\Delta Ct < -5$). Circular isoforms show no significant decrease in abundance with RNase R treatment, and in many cases the signal increases (see main text). The absolute Ct for mock-treated RNA is also given, as an indicator of the

comparative abundance of circular and linear isoforms. For *S. pombe*, data shown here is for exponential growth. b) Quantitation of RNase R resistance for two additional species, *Dictyostelium discoideum* and *S. cerevisiae*. Format is the same as a).
(TIF)

Text S1 PCR primers, Sanger sequencing. 1) List of primers used in PCR and qPCR. 2) Sanger sequences of detected circular RNA isoforms.
(DOC)

Table S1 *S. pombe* candidate circular isoforms, data of Marguerat *et al.* All candidate circular isoforms detected in *S. pombe* from the data of Marguerat *et al.* [19], including readcounts and predicted circle sizes.
(TXT)

Table S2 *S. pombe* candidate circular isoforms, data of Awan *et al.* All candidate circular isoforms detected in *S. pombe* from the data of Awan *et al.* [28], including the gene identifiers, donor and acceptor exons, read sequence and junctional offset, and sample number.
(TXT)

Acknowledgments

We thank Joseph DeRisi and Danny Ebert (UCSF) for providing us with RNA and DNA from *P. falciparum*, and Doug Robinson and Cathy Kabacoff (Johns Hopkins) for providing us with *Dictyostelium* cells.

Author Contributions

Conceived and designed the experiments: PLW YB MCY SBP POB JS. Performed the experiments: PLW YB MCY SPB MNO JS. Analyzed the data: PLW YB MCY SPB POB JS. Contributed reagents/materials/analysis tools: GJH JD. Wrote the paper: PLW POB JS.

References

1. Hsu MT, Coca-Prados M (1979) Electron microscopic evidence for the circular form of RNA in the cytoplasm of eukaryotic cells. Nature 280: 339–340.
2. Nigro JM, Cho KR, Fearon ER, Kern SE, Ruppert JM, et al. (1991) Scrambled exons. Cell 64: 607–613.
3. Cocquerelle C, Mascrez B, Hetuin D, Bailleul B (1993) Mis-splicing yields circular RNA molecules. FASEB J 7: 155–160.
4. Bailleul B (1996) During in vivo maturation of eukaryotic nuclear mRNA, splicing yields excised exon circles. Nucleic Acids Res 24: 1015–1019.
5. Hansen TB, Wiklund ED, Bramsen JB, Villadsen SB, Statham AL, et al. (2011) miRNA-dependent gene silencing involving Ago2-mediated cleavage of a circular antisense RNA. EMBO J 30: 4414–4422. doi: 10.1038/emboj.2011.359.
6. Houseley JM, Garcia-Casado Z, Pascual M, Paricio N, O'Dell KM, et al. (2006) Noncanonical RNAs from transcripts of the Drosophila muscleblind gene. J Hered 97: 253–260. doi: 10.1093/jhered/esj037.
7. Burd CE, Jeck WR, Liu Y, Sanoff HK, Wang Z, et al. (2010) Expression of linear and novel circular forms of an INK4/ARF-associated non-coding RNA correlates with atherosclerosis risk. PLoS Genet 6: e1001233. doi: 10.1371/journal.pgen.1001233.
8. Lamm AT, Stadler MR, Zhang H, Gent JI, Fire AZ (2011) Multimodal RNA-seq using single-strand, double-strand, and CircLigase-based capture yields a refined and extended description of the C. elegans transcriptome. Genome Res 21: 265–275. doi: 10.1101/gr.108845.110.
9. Salzman J, Gawad C, Wang PL, Lacayo N, Brown PO (2012) Circular RNAs are the predominant transcript isoform from hundreds of human genes in diverse cell types. PLoS One 7: e30733. doi: 10.1371/journal.pone.0030733.
10. Salzman J, Chen RE, Olsen MN, Wang PL, Brown PO (2013) Cell-type specific features of circular RNA expression. PLoS Genet 9: e1003777. doi: 10.1371/journal.pgen.1003777.
11. Jeck WR, Sorrentino JA, Wang K, Slevin MK, Burd CE, et al. (2013) Circular RNAs are abundant, conserved, and associated with ALU repeats. RNA 19: 141–157. doi: 10.1261/rna.035667.112.
12. Memczak S, Jens M, Elefsinioti A, Torti F, Krueger J, et al. (2013) Circular RNAs are a large class of animal RNAs with regulatory potency. Nature 495: 333–338. doi: 10.1038/nature11928.
13. Irimia M, Maeso I, Roy SW, Fraser HB (2013) Ancient cis-regulatory constraints and the evolution of genome architecture. Trends Genet 29: 521–528. doi: 10.1016/j.tig.2013.05.008.
14. Csuros M, Rogozin IB, Koonin EV (2011) A detailed history of intron-rich eukaryotic ancestors inferred from a global survey of 100 complete genomes. PLoS Comput Biol 7: e1002150. doi: 10.1371/journal.pcbi.1002150.
15. Shao X, Shepelev V, Fedorov A (2006) Bioinformatic analysis of exon repetition, exon scrambling and trans-splicing in humans. Bioinformatics 22: 692–698. doi: 10.1093/bioinformatics/bti795.
16. Sijen T, Fleenor J, Simmer F, Thijssen KL, Parrish S, et al. (2001) On the role of RNA amplification in dsRNA-triggered gene silencing. Cell 107: 465–476.
17. Vincent HA, Deutscher MP (2006) Substrate recognition and catalysis by the exoribonuclease RNase R. J Biol Chem 281: 29769–29775. doi: 10.1074/jbc.M606744200.
18. Zaphiropoulos PG (1996) Circular RNAs from transcripts of the rat cytochrome P450 2C24 gene: correlation with exon skipping. Proc Natl Acad Sci U S A 93: 6536–6541.
19. Marguerat S, Schmidt A, Codlin S, Chen W, Aebersold R, et al. (2012) Quantitative analysis of fission yeast transcriptomes and proteomes in proliferating and quiescent cells. Cell 151: 671–683. doi: 10.1016/j.cell.2012.09.019.
20. Yanagida M (2009) Cellular quiescence: are controlling genes conserved? Trends Cell Biol 19: 705–715. doi: 10.1016/j.tcb.2009.09.006.
21. Hossain MA, Rodriguez CM, Johnson TL (2011) Key features of the two-intron Saccharomyces cerevisiae gene SUS1 contribute to its alternative splicing. Nucleic acids research 39: 8612–8627. doi: 10.1093/nar/gkr497.
22. Dubin RA, Kazmi MA, Ostrer H (1995) Inverted repeats are necessary for circularization of the mouse testis Sry transcript. Gene 167: 245–248.
23. Irimia M, Roy SW (2008) Evolutionary convergence on highly-conserved 3′ intron structures in intron-poor eukaryotes and insights into the ancestral eukaryotic genome. PLoS Genet 4: e1000148. doi: 10.1371/journal.pgen.1000148.
24. Robberson BL, Cote GJ, Berget SM (1990) Exon definition may facilitate splice site selection in RNAs with multiple exons. Mol Cell Biol 10: 84–94.
25. Romfo CM, Alvarez CJ, van Heeckeren WJ, Webb CJ, Wise JA (2000) Evidence for splice site pairing via intron definition in Schizosaccharomyces pombe. Mol Cell Biol 20: 7955–7970.
26. Moldon A, Malapeira J, Gabrielli N, Gogol M, Gomez-Escoda B, et al. (2008) Promoter-driven splicing regulation in fission yeast. Nature 455: 997-1000. doi: 10.1038/nature07325.
27. Ram O, Ast G (2007) SR proteins: a foot on the exon before the transition from intron to exon definition. Trends Genet 23: 5–7. doi: 10.1016/j.tig.2006.10.002.
28. Awan AR, Manfredo A, Pleiss JA (2013) Lariat sequencing in a unicellular yeast identifies regulated alternative splicing of exons that are evolutionarily conserved with humans. Proc Natl Acad Sci U S A 110: 12762–12767. doi: 10.1073/pnas.1218353110.
29. De Conti L, Baralle M, Buratti E (2013) Exon and intron definition in pre-mRNA splicing. Wiley Interdiscip Rev RNA 4: 49–60. doi: 10.1002/wrna.1140.
30. Singh PK, Kanodia S, Dandin CJ, Vijayraghavan U, Malhotra P (2012) Plasmodium falciparum Prp16 homologue and its role in splicing. Biochim Biophys Acta 1819: 1186–1199. doi: 10.1016/j.bbagrm.2012.08.014.
31. Zhang X, Tolzmann CA, Melcher M, Haas BJ, Gardner MJ, et al. (2011) Branch point identification and sequence requirements for intron splicing in Plasmodium falciparum. Eukaryot Cell 10: 1422–1428. doi: 10.1128/EC.05193-11.
32. Sorber K, Dimon MT, DeRisi JL (2011) RNA-Seq analysis of splicing in Plasmodium falciparum uncovers new splice junctions, alternative splicing and splicing of antisense transcripts. Nucleic Acids Res 39: 3820–3835. doi: 10.1093/nar/gkq1223.
33. Caldas C, So CW, MacGregor A, Ford AM, McDonald B, et al. (1998) Exon scrambling of MLL transcripts occur commonly and mimic partial genomic duplication of the gene. Gene 208: 167–176.
34. Hansen TB, Jensen TI, Clausen BH, Bramsen JB, Finsen B, et al. (2013) Natural RNA circles function as efficient microRNA sponges. Nature 495: 384–388. doi: 10.1038/nature11993.
35. Baum J, Papenfuss AT, Mair GR, Janse CJ, Vlachou D, et al. (2009) Molecular genetics and comparative genomics reveal RNAi is not functional in malaria parasites. Nucleic Acids Res 37: 3788–3798. doi: 10.1093/nar/gkp239.
36. Drinnenberg IA, Weinberg DE, Xie KT, Mower JP, Wolfe KH, et al. (2009) RNAi in budding yeast. Science 326: 544–550. doi: 10.1126/science.1176945.
37. Nam K, Lee G, Trambley J, Devine SE, Boeke JD (1997) Severe growth defect in a Schizosaccharomyces pombe mutant defective in intron lariat degradation. Mol Cell Biol 17: 809–818.
38. Eichinger L, Pachebat JA, Glockner G, Rajandream MA, Sucgang R, et al. (2005) The genome of the social amoeba Dictyostelium discoideum. Nature 435: 43–57. doi: 10.1038/nature03481.
39. Collart MA, Oliviero S (2001) Preparation of yeast RNA. Curr Protoc Mol Biol Chapter 13: Unit 13.12. doi: 10.1002/0471142727.mb1312s23.
40. Li Y, Dai X, Cheng Y, Zhao Y (2011) NPY genes play an essential role in root gravitropic responses in Arabidopsis. Mol Plant 4: 171–179. doi: 10.1093/mp/ssq052.
